# On why cancer cells require a great amount of glucose

**DOI:** 10.1002/qub2.70025

**Published:** 2025-12-05

**Authors:** Xuechen Mu, Aoran Liu, Rui Shi, Long Xu, Zhenyu Huang, Ye Zhang, Ying Xu

**Affiliations:** ^1^ SUSTech Homeostatic Medicine Institute School of Medicine Southern University of Science and Technology Shenzhen Guangdong China; ^2^ Department of Human Cell Biology and Genetics SUSTech Homeostatic Medicine Institute School of Medicine Southern University of Science and Technology Shenzhen Guangdong China; ^3^ Key University Laboratory of Metabolism and Health of Guangdong Southern University of Science and Technology Shenzhen Guangdong China; ^4^ College of Mathematics Jilin University Changchun Jilin China; ^5^ Cancer Institute The First Hospital of China Medical University Shenyang Liaoning China; ^6^ College of Chemistry Jilin University Changchun Jilin China; ^7^ College of Computer Science and Technology Jilin University Changchun Jilin China

**Keywords:** cancer metabolism, cancer proliferation, Fenton reaction, nucleotide biosynthesis, pH homeostasis

## Abstract

The traditional thinking has been that cancer cells require a great amount of glucose to support their rapid growth, but the reality may be different. We have previously demonstrated that all cancer cells in The Cancer Genome Atlas harbor persistent Fenton reactions in their cytosol, which generate ${\text{OH}}^{-}$ and ultimately kill the cells by alkalosis if not neutralized timely. Here, we present data to show that (1) cancer cells uptake large amounts of glucose to produce sufficient levels of $H^{+}$ions to keep the cytosolic pH stable, hence keeping the cells viable; (2) *de novo* nucleotide biosynthesis represents the predominant acidifying pathway and gets on average the largest allocation of glucose metabolic flux among the 19 cancer types investigated; and (3) although the $H^{+}$ions produced by nucleotide biosynthesis and other acidifiers keep the cells alive, the synthesized nucleotides drive cancerous cell proliferation. Taken together, it is not that cancerous cell division requires high levels of glucose imports, instead it is the life‐saving nucleotide syntheses that drive cell division. Understanding this causal relationship correctly is significant since it explains why cancers depend so heavily on glucose but not on other nutrients. More importantly, this realization may lead to fundamentally novel and more effective ways to treat cancer.

## 1 INTRODUCTION

It has long been known that cancer cells consume considerably more glucose than their normal matching cells, earning them the “sweet tooth” nickname [1]. Although this observation provides an opportunity for cancer detection via positron emission tomography/computed tomography, the true reason for this behavior remains an unsettled issue. A widely held belief is that increased glucose uptake is required to support the rapid cancer cell proliferation [2], but the drivers of cancer’s proliferation and its rate remain a controversy, to say the least, or a mystery to be more accurate as cancer researchers are yet to pinpoint which genes or gene functions are responsible for the onset of cancer [3], which led to the discussion of cancer’s heterogeneity [4]. It has been observed that limiting the glucose supply can kill cancer cells but not normal dividing cells, as long as they are fed with other nutrients such as proteins and lipids [5]. This strongly suggests that there are other, possibly fundamental, reasons for the increased glucose utilization by cancer. The importance of addressing this issue lies in that it will not only lead to new understanding about cancer biology but also shed new lights on cancer treatment through restraining glucose supply.

It has been well documented that cancer tissue cells have higher levels of intracellular pH than their matching normal cells, elevating from a neutral pH to around 7.4 [6]. One popular view has been that this is the result of persistent activation of the ${\text{Na}}^{+}$/$H^{+}$ exchanger, NHE1, which exports intracellular $H^{+}$ and imports extracellular ${\text{Na}}^{+}$ [7]. We have previously shown that this is not possible for cancer cells based on a thermodynamic analysis [8]. To further investigate the possible cause of cancer cells’ alkaline intracellular pH, we have studied the implications of Fenton reactions (FR), which have been known to take place in cancer cells [9].

We have demonstrated that all cancer tissues in The Cancer Genome Atlas (TCGA) harbor persistent FR in their cytosol [8, 9],

$$H_{2}O_{2}+\cdot O_{2}\;\overset{{Fe}^{2+}}{\to }\;{\text{OH}}^{-}+\cdot \text{OH}+O_{2},$$

which continuously produce ${\text{OH}}^{-}$, alkalizing the intracellular space as long as inflammation is present, namely, the innate immunity is active in $H_{2}O_{2}$ and $\cdot O_{2}$ production. It is well established that cellular pH is a fundamental property in cell biology as it affects protein folding, the localization of charged molecules such as proteins, enzymatic reaction rates, and some higher‐level properties [10]. Hence, it is essential to keep its stability for the cells’ viability. All cells each maintain an intracellular pH buffer [11] and a suite of acid/base (co‐)transporters to keep the pH stable, when acid‐/base‐producing metabolic fluxes may affect the intracellular pH. However, all these capabilities have relatively small capacities and could not be used to cope with persistent alkalization in a sustained manner, as otherwise it may violate cells’ electroneutrality or other ionic homeostasis [12]. Hence, to deal with such alkalization by FR, cells must utilize other mechanisms to keep the pH stable.

We have previously demonstrated [8, 9, 13] that (1) around 50 acidifying reprogrammed metabolisms (RMs) are employed by different cancer types to keep the intracellular pH stable; (2) a few such RMs are highly conserved and utilized by all cancer tissue samples in TCGA, such as *de novo* synthesis of (dioxy)nucleotides, glycosylation, and ganglioside/sialic acid synthesis (Note: the synthesis of each purine produces 8–9 net protons and a pyrimidine 3–5 protons [14]), whereas other RMs tend to be less conserved and used only by some cancer (sub)types, such as simultaneous triglyceride synthesis and degradation, *de novo* synthesis of serine, and proline degradation; and (3) different cancer (sub)types each employ a distinct subset of these RMs, which gives rise to both the common characteristics shared by all cancers, such as persistent cell division and cell migration given time, and distinguishing properties of individual (sub)types of cancers, such as drug‐sensitive versus drug‐resistant cancers and prone versus less prone to metastases.

**FIGURE 1:**
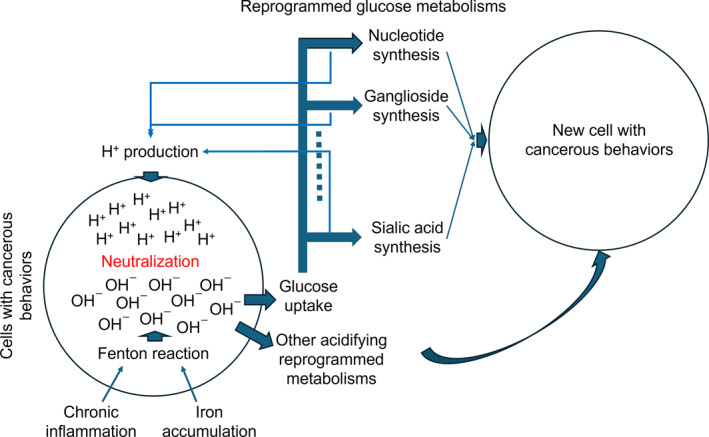
A schematic illustration of how glucose influx and metabolisms drive cancerous behaviors, such as cell proliferation and migration while keeping the intracellular pH stable and the cells viable.

We present a computational study here to demonstrate that it is the level of glucose import, dictated by the level of FR, which is largely responsible for the proliferation rate of cancer, not the other way around as generally believed. We will (1) first present a computational analysis of the distribution of the imported glucose across different downstream pathways; (2) then show the levels of $H^{+}$ production by these glucose metabolisms toward keeping the pH stable; and (3) illustrate how these metabolisms lead to some of the key characteristics of cancer, in addition to keeping the cells alive. Figure 1 shows the basic idea of our model.

Our study has significantly extended the published studies on cancer glucose metabolisms [15, 16, 17] in both the scope and the depth. Specifically, our study has (1) considered substantially more glucose metabolic pathways than those considered by previous studies, including now glycosylation, ganglioside synthesis, and sialic acid synthesis among others; (2) quantitatively estimated the fluxes from the imported glucose to each of the downstream pathways, hence providing an accurate estimate of how the glucose‐derived carbons are distributed across these pathways in each cancer type and stage under study; (3) estimated the rate of H^+^ production by each of the glucose metabolic pathways and established a quantitative relationship between the total rate of H^+^ production by all these pathways and the rate of OH^−^ production by FR defined earlier; and (4) provided a well‐supported explanation of why cancer cells tend to consume considerably more glucose than the matching normal tissues.

## 2 RESULTS

### 2.1 Glucose metabolisms in cancer

**FIGURE 2:**
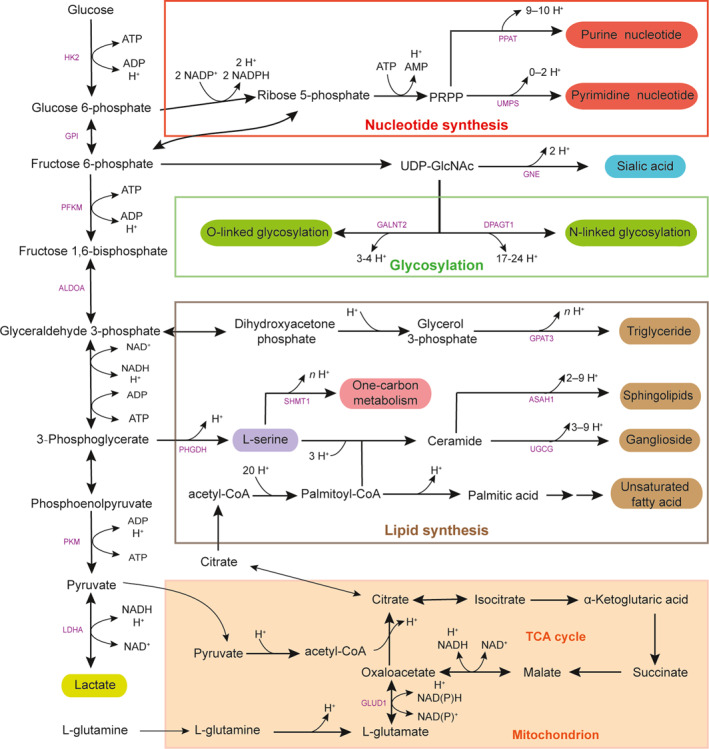
Major metabolic pathways from glucose toward production of (1) nucleotides, (2) lactate, (3) TCA cycle, (4) lipids (unsaturated fatty acid, triglyceride, and sphingolipids), (5) ganglioside + sialic acid synthesis, (6) glycosylation, (7) serine, and (8) one‐carbon metabolism, each of which plays important roles throughout cancer’s evolution. TCA, tricarboxylic acid.

We conducted a comprehensive analysis of glucose metabolism to construct a glucose‐centric metabolic network in cancer, integrating data from the Kyoto Encyclopedia of Genes and Genomes (KEGG) [18] and HumanCyc [19] with gene‐expression profiles from cancer tissues across 19 cancer types (see Section 5). For each cancer type, we identified all expressed enzyme‐encoding genes, which collectively define an acyclic metabolic network originating from glucose uptake. This process was replicated across all cancer types. Figure 2 presents a consensus model of the glucose metabolic network, comprising only those pathways exhibiting upregulation in at least two stages (stages 1–4) across the 19 cancer types. Relevant expression data for these pathways are provided in Table S1.

To quantify the distribution of imported glucose across these pathways, we estimated flux allocations for all tissue samples across each stage and cancer type. The approach involved parameterizing a quantitative flux balance model for each pathway in the network depicted in Figure 2. For each glucose metabolic pathway considered, we established a biologically informed upper bound on the maximal flux by integrating pathway activity scores—calculated via Single‐Sample Gene Set Enrichment Analysis (ssGSEA) [20]—and the estimated catalytic efficiency constants ($\frac{k_{\text{cat}}}{K_{m}}$) of the enzymes involved in the pathway, thereby defining pathway‐specific capacity constraints [21]. These constraints were then incorporated into the glucose metabolic network, and the glucose‐derived carbon fluxes were predicted by solving a quadratic programming (QP) problem that optimizes a weighted objective function. This optimization was subject to two principal constraints: (1) mass balance at every branch point, ensuring that the total influx equals the total efflux, and (2) the estimated fluxes are upper‐bounded by their estimated capacities. Details are provided in Section 5.

To ensure accurate flux estimation, we first estimated cancer‐specific $\frac{k_{\text{cat}}}{K_{m}}$ values and then used these as pathway‐specific upper bounds in subsequent flux calculation. For each cancer, gene‐level fold changes and *p*‐values were calculated by comparing transcriptomic data from cancer samples against the corresponding normal tissues; these statistics were used as weights and multiplied by normalized gene expression to construct a reaction activity proxy matrix for each pathway. Under the pseudo‐steady‐state assumption, this activity matrix was combined with the prior catalytic efficiency information from BRENDA [22] and the CatPred deep‐learning predictor [23], and a regularized nonnegative least squares optimization (NNLS) was performed to obtain $\frac{k_{\text{cat}}}{K_{m}}$ estimates. This approach ensures that the resulting $\frac{k_{\text{cat}}}{K_{m}}$ values reflect both the catalytic potential of enzymes and their actual expression levels in each cancer tissue across eight glucose metabolic pathways. To validate these estimates, reactions were mapped to their primary Enzyme Commission (EC) classifications [24] and Kruskal–Wallis *H*‐tests [25] were applied, revealing significant differences in median $\frac{k_{\text{cat}}}{K_{m}}$ values across EC classes (*p* < 0.05) in all 19 cancer types. These findings reveal that the estimated $\frac{k_{\text{cat}}}{K_{m}}$ values vary across enzyme functional categories, reflecting the diversity of catalytic efficiency tailored to cancer‐specific metabolic demands. The robustness of these distinctions, confirmed by the nonparametric Kruskal–Wallis *H*‐test which accommodates non‐normal distributions typical in biological data, is visualized in Figure S1 as logarithmic boxplots. These results underscore the biological validity of the estimated $\frac{k_{\text{cat}}}{K_{m}}$ values and reveal expression patterns that are consistent with cancer‐specific metabolic reprogramming.

**FIGURE 3:**
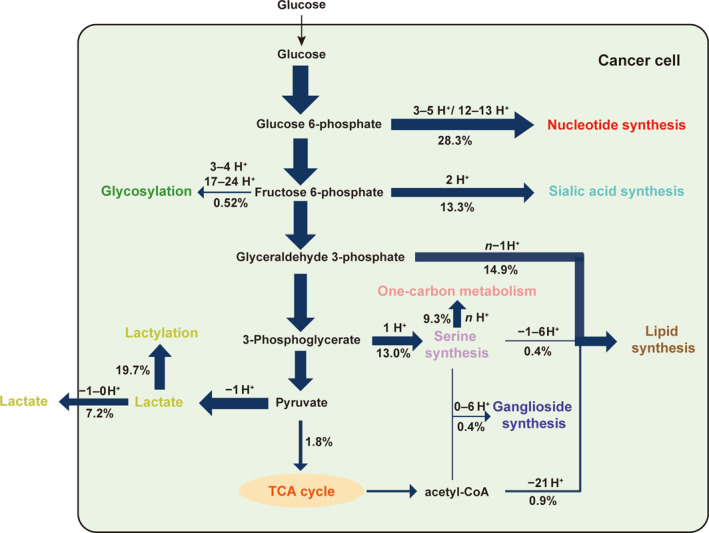
An illustrative example of glucose metabolic pathways, where the thickness of each arrow represents the typical proportion of the level of the glucose‐derived carbon flux across different cancer types.

Table 1 details the estimated fractional allocation of imported glucose to each glucose metabolic pathway across all stages and cancer types. Figure 3 provides an illustrative overview of the average glucose distribution across pathways for multiple cancer types. Our flux analysis reveals that *de novo* nucleotide synthesis accounts for the largest fraction of imported glucose (as detailed in Table 1). The pathways of nucleotide synthesis, lactate production, and lipid synthesis rank as the top three glucose‐consuming pathways across all 19 cancer types. A regression analysis of total glucose importer expression against the expression levels of these three pathways yielded high $R^{2}$ values, as presented in Table 2, confirming their predominant utilization of imported glucose across all cancer types.

**TABLE 1:** The estimated percentages of glucose flux distribution across eight glucose pathways across different stages of 19 cancer types.

Metabolic process	Stage	BLCA	BRCA	CESC	COAD	ESCA	HNSC	KICH	KIRC	KIRP	LIHC	LUAD	LUSC	OV	PAAD	READ	SKCM	STAD	THCA	UCEC
TCA	S1	0.80	1.25	0.83	0.99	1.31	0.86	9.16	0.05	0.74	0.75	0.90	1.03	0.98	0.99	1.31	0.86	8.95	0.78	0.71
	S2	0.77	1.48	0.85	1.05	1.13	1.05	12.30	0.04	0.75	0.76	0.79	0.96	0.86	0.88	1.35	0.96	8.62	0.73	0.77
	S3–4	0.79	1.31	0.70	1.08	1.23	0.84	8.35	0.04	0.77	0.79	0.75	0.94	0.83	1.10	1.31	0.88	9.30	0.89	0.80
Ganglioside synthesis	S1	0.36	0.54	0.23	0.48	0.97	0.41	0.30	0	0.43	0.37	0.35	0.33	0.22	0.40	0.50	0.31	0.28	0.48	0.36
	S2	0.39	0.59	0.19	0.44	0.51	0.42	0.24	0	0.37	0.35	0.35	0.36	0.22	0.35	0.52	0.45	0.22	0.54	0.36
	S3–4	0.40	0.63	0.28	0.54	0.65	0.47	0.36	0.01	0.47	0.43	0.34	0.31	0.34	0.43	0.61	0.43	0.42	0.56	0.45
Glycosylation	S1	0.45	0.78	0.38	0.72	0.65	0.45	0.70	0.03	0.54	0.41	0.43	0.55	0.55	0.49	0.78	0.32	0.47	0.57	0.44
	S2	0.58	0.67	0.36	0.66	0.67	0.52	0.69	0.02	0.57	0.45	0.46	0.56	0.49	0.52	0.77	0.57	0.51	0.57	0.44
	S3–4	0.47	0.74	0.38	0.57	0.68	0.46	0.64	0.01	0.45	0.40	0.47	0.56	0.53	0.55	0.64	0.56	0.51	0.56	0.49
Lactate production	S1	30.16	49.23	28.98	44.98	40.12	35.09	0.72	5.76	34.73	9.47	10.11	32.73	11.91	28.57	40.68	33.15	30.30	13.80	34.67
	S2	32.78	51.18	26.14	42.48	40.65	32.13	0.59	5.01	27.76	10.18	10.56	30.44	15.68	27.72	41.08	31.78	31.14	12.04	27.32
	S3–4	31.05	50.67	27.94	45.68	41.67	35.74	0.64	4.19	31.96	10.49	9.68	33.94	12.14	26.03	46.85	30.52	33.53	13.82	33.74
Nucleotide synthesis	S1	46.52	19.07	16.98	12.55	29.36	34.90	52.86	1.90	47.85	27.90	28.19	15.43	33.59	24.36	15.80	56.92	11.73	41.59	19.03
	S2	43.38	19.56	18.89	14.36	28.99	35.20	54.16	1.61	56.13	27.74	25.20	14.37	31.40	19.87	17.77	54.19	12.33	42.08	23.39
	S3–4	46.23	21.44	18.24	12.90	28.30	33.57	48.87	1.65	51.19	32.73	27.64	15.97	35.20	22.44	14.96	52.78	13.98	37.38	18.90
One carbon metabolism	S1	3.40	6.42	19.56	4.77	5.22	0.63	25.10	2.25	0.71	0.53	2.79	15.89	23.36	20.89	4.71	3.02	21.95	4.47	14.82
	S2	3.74	5.58	20.93	4.31	5.64	0.60	21.95	0	0.68	0.54	2.96	18.00	23.44	21.85	4.47	3.56	22.80	3.65	16.60
	S3–4	2.98	4.26	18.44	4.05	4.63	0.49	28.96	1.85	0.62	0.46	2.48	15.17	20.69	17.96	4.19	4.07	17.64	4.03	13.78
Serine synthesis	S1	1.38	2.54	6.65	1.89	2.29	0.51	8.48	0.74	0.53	0.42	1.16	5.52	7.92	7.18	1.92	1.20	7.48	1.83	5.16
	S2	1.52	2.28	7.08	1.73	2.21	0.50	7.44	0	0.50	0.43	1.24	6.22	7.94	7.49	1.85	1.47	7.74	1.59	5.73
	S3–4	1.25	1.87	6.31	1.70	1.95	0.48	9.74	0.62	0.52	0.44	1.06	5.27	7.08	6.24	1.79	1.62	6.11	1.72	4.87
Sialic acid synthesis	S1	10.10	17.52	15.07	24.17	17.54	13.10	0.73	2.73	12.46	2.13	10.57	13.92	14.47	10.96	31.64	3.20	17.31	13.20	18.02
	S2	10.00	15.82	13.96	24.95	17.86	14.13	0.64	3.03	11.60	2.31	12.31	13.85	12.48	14.41	29.49	5.13	15.09	15.82	19.06
	S3–4	10.23	16.09	16.44	23.40	18.39	13.39	0.72	2.23	12.17	2.37	10.98	13.80	15.61	17.67	26.83	7.36	16.86	15.87	18.96
Lipid synthesis	S1	6.84	2.64	11.32	9.45	2.54	14.05	1.94	86.53	2.01	58.01	45.51	14.60	6.99	6.16	2.65	1.02	1.54	23.27	6.80
	S2	6.84	2.85	11.59	10.01	2.34	15.45	2.00	90.30	1.65	57.25	46.12	15.23	7.48	6.90	2.71	1.89	1.54	22.98	6.32
	S3–4	6.60	2.99	11.27	10.07	2.48	14.56	1.72	89.40	1.85	51.91	46.60	14.04	7.58	7.57	2.81	1.78	1.65	25.46	8.00

Abbreviations: BLCA, bladder urothelial carcinoma; BRCA, breast invasive carcinoma; CESC, cervical squamous cell carcinoma and endocervical adenocarcinoma; COAD, colon adenocarcinoma; ESCA, esophageal carcinoma; HNSC, head and neck squamous cell carcinoma; KICH, kidney chromophobe; KIRC, kidney renal clear cell carcinoma; KIRP, kidney renal papillary cell carcinoma; LIHC, liver hepatocellular carcinoma; LUAD, lung adenocarcinoma; LUSC, lung squamous cell carcinoma; OV, ovarian serous cystadenocarcinoma; PAAD, pancreatic adenocarcinoma; READ, rectum adenocarcinoma; SKCM, skin cutaneous melanoma; STAD, stomach adenocarcinoma; TCA, tricarboxylic acid; THCA, thyroid carcinoma; UCEC, uterine corpus endometrial carcinoma.

**TABLE 2:** The estimated percentage of total glucose consumption by the top three glucose metabolic pathways out of the total glucose import across different stages of 19 cancer types.

Cancer type	Stage I	Stage II	Stage III and IV
BLCA	0.6365 ($9.43\times 10^{-17}$)	0.7655 ($6.02\times 10^{-26}$)	0.5847 ($1.09\times 10^{-36}$)
BRCA	0.8182 ($1.38\times 10^{-48}$)	0.6718 ($2.03\times 10^{-114}$)	0.7619 ($2.10\times 10^{-54}$)
CESC	0.6729 ($4.45\times 10^{-27}$)	0.7947 ($1.13\times 10^{-09}$)	0.8633 ($1.49\times 10^{-11}$)
COAD	0.7062 ($2.68\times 10^{-10}$)	0.7539 ($1.12\times 10^{-18}$)	0.6427 ($4.89\times 10^{-15}$)
ESCA	0.9516 ($5.68\times 10^{-05}$)	0.9061 ($7.54\times 10^{-04}$)	0.9286 ($2.37\times 10^{-02}$)
HNSC	0.7240 ($4.19\times 10^{-62}$)	0.7595 ($5.12\times 10^{-09}$)	0.7288 ($3.84\times 10^{-77}$)
KICH	0.9556 ($5.98\times 10^{-08}$)	0.9501 ($8.05\times 10^{-06}$)	0.9502 ($6.40\times 10^{-06}$)
KIRC	0.7768 ($3.83\times 10^{-63}$)	0.8831 ($3.39\times 10^{-06}$)	0.8794 ($7.18\times 10^{-41}$)
KIRP	0.6924 ($7.90\times 10^{-31}$)	0.9673 ($4.28\times 10^{-05}$)	0.9514 ($7.74\times 10^{-33}$)
LIHC	0.3062 ($1.28\times 10^{-05}$)	0.4832 ($4.67\times 10^{-04}$)	0.6254 ($3.08\times 10^{-08}$)
LUAD	0.6196 ($2.81\times 10^{-46}$)	0.7572 ($3.70\times 10^{-22}$)	0.7393 ($2.03\times 10^{-17}$)
LUSC	0.7226 ($5.39\times 10^{-50}$)	0.7126 ($1.21\times 10^{-27}$)	0.6898 ($1.87\times 10^{-10}$)
OV	0.8144 ($8.57\times 10^{-06}$)	0.9601 ($4.60\times 10^{-07}$)	0.7231 ($4.58\times 10^{-55}$)
PAAD	0.9501 ($2.65\times 10^{-04}$)	0.8521 ($3.09\times 10^{-39}$)	0.9721 ($1.15\times 10^{-02}$)
READ	0.9191 ($2.22\times 10^{-01}$)	0.7240 ($9.15\times 10^{-01}$)	0.7961 ($2.36\times 10^{-04}$)
SKCM	0.9686 ($5.58\times 10^{-03}$)	0.7383 ($8.54\times 10^{-08}$)	0.9475 ($1.43\times 10^{-03}$)
STAD	0.6809 ($2.74\times 10^{-11}$)	0.5538 ($2.97\times 10^{-10}$)	0.5793 ($4.20\times 10^{-25}$)
THCA	0.9165 ($9.90\times 10^{-151}$)	0.9404 ($1.02\times 10^{-13}$)	0.8722 ($6.83\times 10^{-53}$)
UCEC	0.8059 ($1.82\times 10^{-19}$)	0.9560 ($6.89\times 10^{-02}$)	0.7721 ($9.81\times 10^{-05}$)

Abbreviations: BLCA, bladder urothelial carcinoma; BRCA, breast invasive carcinoma; CESC, cervical squamous cell carcinoma and endocervical adenocarcinoma; COAD, colon adenocarcinoma; ESCA, esophageal carcinoma; HNSC, head and neck squamous cell carcinoma; KICH, kidney chromophobe; KIRC, kidney renal clear cell carcinoma; KIRP, kidney renal papillary cell carcinoma; LIHC, liver hepatocellular carcinoma; LUAD, lung adenocarcinoma; LUSC, lung squamous cell carcinoma; OV, ovarian serous cystadenocarcinoma; PAAD, pancreatic adenocarcinoma; READ, rectum adenocarcinoma; SKCM, skin cutaneous melanoma; STAD, stomach adenocarcinoma; THCA, thyroid carcinoma; UCEC, uterine corpus endometrial carcinoma.

**FIGURE 4:**
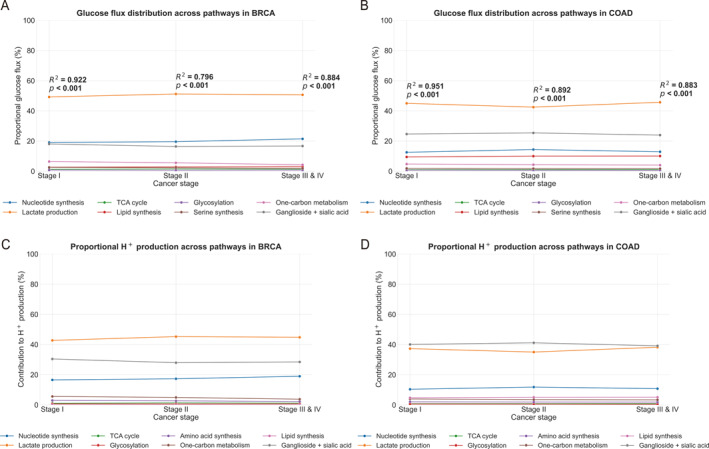
Glucose allocation and $H^{+}$ production in BRCA and COAD. (A) and (B) show the percentage of glucose import allocated to eight metabolic pathways across cancer stages, respectively, whereas (C) and (D) display the corresponding percentage of $H^{+}$ production. BRCA, breast invasive carcinoma; COAD, colon adenocarcinoma.

Table 3 summarizes the flux analysis results, revealing a pan‐cancer trend wherein cancer cells, on average, allocate 28.27% of imported glucose to nucleotide synthesis, 26.87% to the lactate production, and 16.20% to lipid synthesis across all tumor types. Figure 4A, B exemplify the estimated flux distributions across the glucose metabolic pathways for breast cancer (BRCA) and colorectal adenocarcinoma (COAD), with results for the other 17 cancer types provided in Figure S2.

**TABLE 3:** The predicted percentage of glucose allocated to each of the eight glucose metabolic pathways across 19 cancer types.

Metabolic process	Mean flux allocation (%)
Nucleotide synthesis	28.2724
Lactate production	26.8742
Lipid synthesis	16.2043
Ganglioside + sialic acid synthesis	13.7285
Serine synthesis	13.0057
One carbon metabolism	9.2730
TCA	1.7905
Glycosylation	0.5167

Abbreviation: TCA, tricarboxylic acid.

### 2.2 H^+^ ions produced by glucose metabolisms toward keeping intracellular pH stable

We aim to understand what dictates the total glucose import and how the glucose is allocated to different glucose metabolic pathways in cancer. We study it from the perspective of maintaining the stability of the intracellular pH, knowing that (1) all cancer tissue cells harbor persistent FR, which continuously alkalize the intracellular space; and (2) the stability of pH is essential to the viability of the cells. Using the method given in Section 5, we have estimated the percentage of the total production rate of $H^{+}$ by each of the eight glucose metabolic pathways out of that by all these pathways. Figure 4C, D present illustrative examples for BRCA and COAD, with corresponding data for the remaining 17 cancer types provided in Figure S3.

Table 4 lists the percentage of the total $H^{+}$ production rate by each glucose metabolic pathway out of the whole glucose metabolism across all 19 cancer types. From this table, we observed that nucleotide synthesis, lactate production, and ganglioside/sialic acid pathways contributed the largest fractions to the total $H^{+}$ production across most cancer types. Specifically, nucleotide synthesis accounts for 25.81% of the total $H^{+}$ production, 23.69% of lactate production, and 23.26% of ganglioside/sialic acid, based on the mean values across the 19 cancer types. Collectively, these three pathways overwhelmingly dominate $H^{+}$ production among the glucose‐derived metabolic branches in cancer.

**TABLE 4:** Percentages of H^+^ production out of all the H^+^ ions produced by eight glucose metabolic pathways across different stages of 19 cancer types.

Metabolic process	Stage	BLCA	BRCA	CESC	COAD	ESCA	HNSC	KICH	KIRC	KIRP	LIHC	LUAD	LUSC	OV	PAAD	READ	SKCM	STAD	THCA	UCEC
TCA	S1	0.75	1.08	0.72	0.82	1.14	0.78	9.04	0.05	0.67	0.76	0.83	0.91	0.86	0.90	1.01	0.84	7.59	0.72	0.61
	S2	0.72	1.31	0.75	0.86	0.98	0.96	12.19	0.04	0.68	0.77	0.72	0.86	0.77	0.78	1.06	0.93	7.44	0.65	0.66
	S3–4	0.74	1.16	0.61	0.90	1.06	0.77	8.20	0.04	0.70	0.80	0.69	0.84	0.73	0.95	1.06	0.83	7.96	0.79	0.69
Ganglioside + sialic acid synthesis	S1	18.85	30.42	26.35	40.09	30.57	23.87	1.45	5.31	22.62	4.31	19.55	24.77	25.34	19.92	49.00	6.26	29.34	24.10	31.06
	S2	18.70	27.96	24.56	41.16	30.86	25.61	1.27	5.88	21.14	4.65	22.43	24.63	22.24	25.30	46.48	9.93	26.04	28.22	32.40
	S3–4	19.13	28.45	28.46	39.16	31.74	24.37	1.41	4.37	22.13	4.78	20.28	24.62	27.17	30.42	43.27	13.93	28.86	28.32	32.57
Glycosylation	S1	0.42	0.68	0.33	0.60	0.57	0.41	0.69	0.03	0.49	0.41	0.40	0.49	0.48	0.44	0.61	0.31	0.40	0.52	0.38
	S2	0.54	0.60	0.32	0.54	0.58	0.47	0.68	0.02	0.52	0.45	0.42	0.50	0.44	0.45	0.61	0.56	0.44	0.51	0.37
	S3–4	0.44	0.65	0.33	0.48	0.59	0.41	0.63	0.01	0.41	0.40	0.44	0.50	0.46	0.48	0.52	0.53	0.44	0.50	0.42
Lactate production	S1	28.15	42.74	25.34	37.30	34.95	31.99	0.71	5.60	31.51	9.57	9.36	29.13	10.42	25.96	31.51	32.41	25.68	12.60	29.89
	S2	30.65	45.24	22.98	35.04	35.12	29.11	0.59	4.87	25.29	10.25	9.63	27.06	13.97	24.33	32.37	30.78	26.87	10.74	23.22
	S3–4	29.03	44.80	24.18	38.24	35.95	32.51	0.63	4.09	29.05	10.61	8.95	30.27	10.57	22.41	37.78	28.88	28.70	12.06	28.98
Nucleotide synthesis	S1	43.42	16.55	14.84	10.40	25.57	31.81	52.17	1.85	43.43	28.19	26.08	13.74	29.41	22.13	12.24	55.65	9.94	37.97	16.41
	S2	40.56	17.29	16.62	11.84	25.05	31.89	53.68	1.56	51.14	27.94	22.96	12.77	27.97	17.44	14.00	52.47	10.64	37.52	19.88
	S3–4	43.21	18.96	15.78	10.80	24.42	30.54	47.96	1.61	46.52	33.10	25.53	14.24	30.63	19.32	12.07	49.94	11.97	33.34	16.23
One carbon metabolism	S1	3.18	5.58	17.10	3.96	4.55	0.57	24.78	2.19	0.64	0.53	2.58	14.14	20.45	18.98	3.65	2.95	18.61	4.08	12.77
	S2	3.50	4.93	18.41	3.56	4.87	0.55	21.76	0	0.62	0.54	2.70	16.00	20.89	19.18	3.53	3.44	19.67	3.25	14.11
	S3–4	2.79	3.77	15.96	3.39	3.99	0.45	28.43	1.81	0.57	0.47	2.29	13.53	18.01	15.46	3.38	3.85	15.10	3.60	11.84
Serine synthesis	S1	1.72	2.94	7.76	2.09	2.66	0.62	11.15	0.96	0.64	0.56	1.43	6.55	9.25	8.69	1.98	1.57	8.45	2.23	5.93
	S2	1.89	2.68	8.30	1.91	2.54	0.60	9.83	0	0.61	0.58	1.50	7.38	9.44	8.76	1.95	1.89	8.90	1.89	6.49
	S3–4	1.56	2.21	7.28	1.90	2.24	0.58	12.75	0.80	0.63	0.59	1.31	6.26	8.22	7.17	1.93	2.04	6.98	2.05	5.58
Lipid synthesis	S1	3.52	0	7.55	4.75	0	9.95	0	84.00	0	55.65	39.76	10.26	3.79	2.97	0	0	0	17.79	2.95
	S2	3.44	0	8.07	5.08	0	10.81	0	87.64	0	54.82	39.64	10.81	4.29	3.76	0	0	0	17.22	2.86
	S3–4	3.11	0	7.40	5.13	0	10.36	0	87.26	0	49.26	40.51	9.73	4.22	3.80	0	0	0	19.34	3.69

Abbreviations: BLCA, bladder urothelial carcinoma; BRCA, breast invasive carcinoma; CESC, cervical squamous cell carcinoma and endocervical adenocarcinoma; COAD, colon adenocarcinoma; ESCA, esophageal carcinoma; HNSC, head and neck squamous cell carcinoma; KICH, kidney chromophobe; KIRC, kidney renal clear cell carcinoma; KIRP, kidney renal papillary cell carcinoma; LIHC, liver hepatocellular carcinoma; LUAD, lung adenocarcinoma; LUSC, lung squamous cell carcinoma; OV, ovarian serous cystadenocarcinoma; PAAD, pancreatic adenocarcinoma; READ, rectum adenocarcinoma; SKCM, skin cutaneous melanoma; STAD, stomach adenocarcinoma; TCA, tricarboxylic acid; THCA, thyroid carcinoma; UCEC, uterine corpus endometrial carcinoma.

Now our question is: what percentage of the O$H^{-}$ions produced by cytosolic FR is naturalized by $H^{+}$ions produced by the eight glucose pathways in each of the 19 cancer types. To quantify this, we have applied a two‐step approach. First, we used a partial least squares (PLS) regression to reduce the high dimensionality of the expression data of genes involved in both FR and the eight glucose metabolic pathways to one‐dimensional scores. Then, we performed a multivariate linear regression to model the relationship between these scores, with the coefficient of determination $R^{2}$ serving as a measure of explanatory power.

The calculation results are given in Figure S5 across all 19 cancer types. Based on these results, we conclude that a vast majority of the O$H^{-}$s produced by Fenton reaction is neutralized by $H^{+}$ produced by glucose‐based metabolisms, ranging from 0.821 to 0.978 across all the cancer types; and this is the actual reason for the large amount of glucose consumed by cancer cells.

### 2.3 Key characteristics resulted from reprogrammed glucose metabolisms

It has long been recognized that cancer cells prefer *de novo* synthesizing rather than uptaking nucleotides from circulation [26]. The previous explanation for this odd behavior was that this is to provide a sufficient pool of nucleotides in support of cancer cells’ rapid proliferation [27]. However, this speculation is not supported by the common knowledge that there is no shortage of nucleosides in the blood circulation of cancer patients of any type; and uptake from circulation is significantly faster than *de novo* synthesis from glucose.

Our next question is: how do the cells get rid of the continuously synthesized (dioxy)nucleotides, knowing that the intracellular accumulation of nucleotides will slow‐down the nucleotide synthesis process, hence the $H^{+}$ production process, resulting in progressively increased alkaline stress and ultimately cell death by alkalosis. Direct release of them extracellularly is not a feasible option since each nucleotide carries a negative charge and their continuous release will violate cells’ electroneutrality, hence resulting in cell death. We have previously formulated a hypothesis: the affected cells synthesize a DNA from these synthesized nucleotides, possibly plus some nucleotide uptake from circulation to make up for the unbalanced populations of A, C, G, and T, which is required for making a DNA [9].

To substantiate this hypothesis, we present two key findings: (1) nucleotide synthesis drives cell cycle progression in cancer, independent of confounding effects from general proliferation signals observed in normal proliferating cells, and (2) the cell proliferation rate is predominantly dictated by the nucleotide synthesis rate. We employed a robust causal inference framework based on Judea Pearl’s Structural Causal Model (SCM) to investigate the causal influence of nucleotide synthesis on cell cycle progression. This analysis addressed two aspects: the directional causality wherein nucleotide synthesis (treatment variable, *T*, quantified via ssGSEA scores of tumor‐specific upregulated genes) drives cell cycle activity (outcome variable, *Y*, measured by normalized $\log_{2}$‐transformed MKI67 expression), distinct from potential reverse causality in normal cells, and the specific contribution of nucleotide synthesis after controlling for general proliferation (confounder variable, *W*). The average treatment effect (ATE) was estimated using a backdoor adjustment via multivariable linear regression ($E\left[Y\right]=\beta_{0}+\beta_{\text{ATE}}\cdot T+\beta_{W}\cdot W+\epsilon$), where $\beta_{\text{ATE}}$ reflects the direct causal impact of *T* on *Y*. Validation through permutation tests yielded highly significant *p*‐values (< 0.0001) for both cancer types, supporting a causal role for nucleotide synthesis.

**FIGURE 5:**
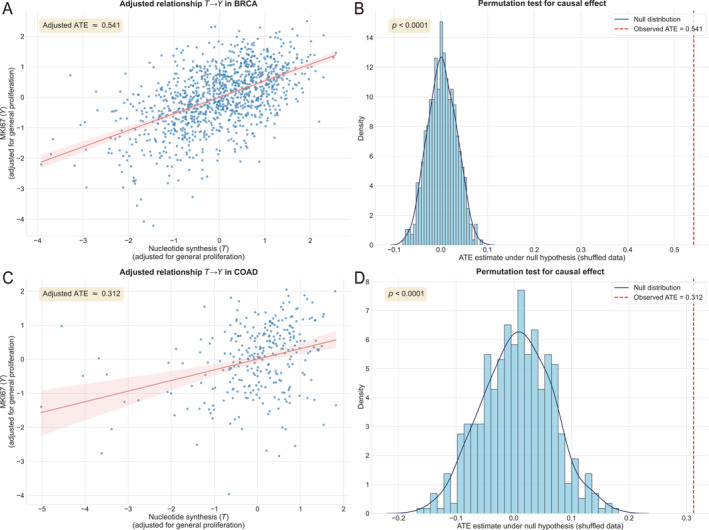
Causal inference analysis of nucleotide synthesis pathway activity on cell cycle progression in BRCA and COAD. (A) Partial regression plot depicting the adjusted relationship between nucleotide synthesis activity (*T*) and cell cycle activity (*Y*, measured by MKI67 expression) after controlling for general proliferation (*W*) in BRCA, with an adjusted ATE of 0.541. (B) Permutation test distribution for BRCA, showing the null distribution of ATE estimates under shuffled data (blue) and the observed ATE (red, 0.541) with a *p*‐value < 0.0001. (C) Partial regression plot for COAD, illustrating the adjusted *T‐Y* relationship with an ATE of 0.312. (D) Permutation test distribution for COAD, displaying the null distribution and observed ATE (red, 0.312) with a *p*‐value < 0.0001. ATE, average treatment effect; BRCA, breast invasive carcinoma; COAD, colon adenocarcinoma.

As depicted in Figure 5, partial regression plots and permutation test distributions for bladder urothelial carcinoma (BLCA) and COAD illustrate these relationships. The adjusted ATE values were 0.541 for BLCA and 0.312 for COAD, both with p<0.0001, indicating a low probability of these effects occurring by chance under the null hypothesis. Confidence intervals in the partial regression plots reinforce the robustness of the positive *T*‐*Y* associations after adjusting for *W*. Sensitivity analyses via refutation tests further validated the stability of these ATE estimates against unmeasured confounders. Collectively, these results affirm that nucleotide synthesis is a primary driver of cell cycle progression in these cancer types, independent of general proliferation effects.

**FIGURE 6:**
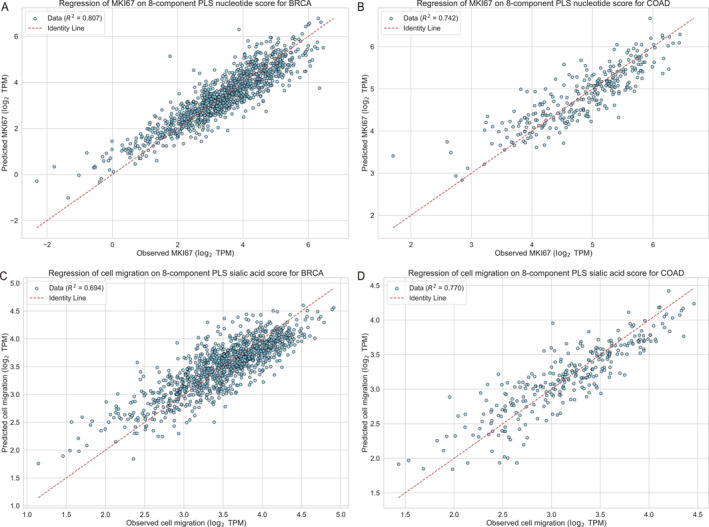
Regression analysis of metabolic signatures in BRCA and COAD. (A and B) The regression of MKI67 expression (log_2_ TPM) on the 8‐component PLS‐derived nucleotide score for BRCA and COAD, respectively, whereas (C and D) present the corresponding regression of observed cell migration (log_2_ TPM) on the 8‐component PLS‐derived sialic acid score. In all panels, blue circles represent individual cancer samples, and the red dashed line denotes the identity line, illustrating the strong correspondence between the metabolic signatures and their associated phenotypes. BRCA, breast invasive carcinoma; COAD, colon adenocarcinoma; PLS, partial least squares; TPM, transcripts per million.

Additionally, we conducted regression analyses of cancer cell cycle rates, assessed via MKI67 expression, against nucleotide synthesis rates calculated using the PLS method (see Figure 6A, B and Section 5). The high $R^{2}$ values for both BRCA and COAD indicate that a substantial proportion of the variance in cell cycle rates is explained by nucleotide synthesis rates. These findings, combined with prior experimental evidence demonstrating that cytosolic FR accelerate cell proliferation [28], strongly support the conclusion that nucleotide synthesis drives cell cycle progression and dictates proliferation rates in cancer cells.

A remaining question is why cancer cells release lactic acid (lactate + $H^{+}$) under alkaline stress. To address this, we performed metabolic flux modeling of lactate production from glucose metabolism, revealing that, on average across 19 tumor types, only 26.64% of the produced lactate is extracellularly released with H^+^ in a 1:1 ratio, as shown in Figure S4. The remaining lactate is retained intracellularly, primarily as protein lactylation, a phenomenon widely observed in cancer cells [29]. This extracellular release accounts for approximately 26.64% of the H^+^ produced by glucose metabolism, whereas intracellular lactic acid serves multiple essential roles in cancer development [30], necessitating its partial release.

Another hallmark of cancer is cell migration and metastasis. We previously hypothesized that the continuous synthesis and surface deployment of sialic acids drive cell migration through a mechanism involving the accumulation of negatively charged sialic acids, which enhances electrostatic repulsion among cells, induces mechanical deformation, and activates epithelial–mesenchymal transition (EMT), a process linked to migration under mechanical compression [31, 32]. To test this, we developed a statistical model correlating cell migration with sialic acid accumulation on cancer cell surfaces.

Figure 6C, D present regression results of observed cell migration scores against predicted values from an 8‐component PLS model of sialic acid deployment genes. The high $R^{2}$ values indicate that sialic acid “deployment scores” effectively explain the variance in cell migration, supporting the prediction that sialic acid accumulation drives this process.

To experimentally validate our integrated model, we explored the metabolic vulnerabilities of cancer cells to glucose restriction under Fenton reaction‐induced alkaline stress. We used two pairs of cancer and normal cell lines: gastric cancer lines (HGC27, AGS) with a normal gastric mucosal epithelial line (GES‐1), and hepatocellular carcinoma lines (LM3, Huh7) with an immortalized normal hepatocyte line (THLE‐2). Following Fenton reaction induction to generate alkaline stress, cells were cultured for 24 h in standard or low‐glucose medium. Cell viability and expression of key nucleotide synthesis genes were assessed via live/dead staining and reverse transcription quantitative PCR (RT‐qPCR), respectively.

Results showed a pronounced glucose dependency in cancer cells. Normal lines (GES‐1 and THLE‐2) exhibited minimal, nonsignificant increases in cell death and slight reductions in nucleotide‐related gene mRNA levels under glucose restriction (Figure S7). In contrast, all cancer lines (HGC27, AGS, LM3, and Huh7) displayed significant increases in cell death and substantial downregulation of nucleotide synthesis gene expression in low‐glucose conditions. These findings confirm that, under alkaline stress, cancer cells are more reliant than normal cells on glucose to counteract alkalosis through metabolic reprogramming and sustain anabolic pathways for proliferation.

## 3 DISCUSSION

We have previously established a new framework for elucidating the drivers and key mechanisms of cancer onset and progression [8, 9, 13, 14]. The core idea of the framework is, as illustrated by Figure 1, that all cancer tissue cells harbor persistent FR in their cytosol, continuously alkalizing the intracellular space. The cells will die from alkalosis if the ${\text{OH}}^{-}$s are not neutralized. Under this increasingly stronger stress, the cells activate a suite of acidifying metabolisms, predominantly glucose metabolisms, to neutralize the ${\text{OH}}^{-}$s, hence keeping the cells viable.

The focus of this study is on the downstream effects of these acidifying glucose metabolisms. We provided strong statistical evidence that the *de novo* nucleotide synthesis, as the major acidifier across all the 19 cancer types, drives cell cycle progression to get rid of the nucleotides via synthesizing them into a DNA and then wrapping it around the positively charged histones, and at the end putting the neutrally charged DNA‐histone complex into a new cell, completing one cycle of cancerous cell division. As the new cells are in the same cancer‐driving microenvironment, they must deal with the same stressor that their parent cells dealt with, hence the persistent cell division.

In this context, we offered a new explanation to a long studied but unsettled behavior of cancer cells: why do cancer cells consume considerably more glucose compared to their matching normal cells. Compared to the traditional thinking, we provided strong statistical evidence that it is the nucleotide synthesis rate dictates the cell‐division rate in cancer cells, which is similar to yeast or other unicellular organisms, where it is the concentrations of nutrients such as nucleotides and sugar that drive cell cycle progression [33]. This requires a fundamental transformation in the cell polarity system via selection of massive mutations in cell‐polarity genes, enabling nucleotide or nucleotide–sugar concentration to drive cell cycle progression as in yeast [34].

In the same context, we offered a new explanation to what may drive cancer cell migration, the first step toward long‐distance metastasis. That is: sialic acids, coupled with ganglioside synthesis and deployed on cancer cell surface, drive cell migration via a previously un‐recognized mechanism—the increasingly stronger cell–cell electrostatic repulsion created by negatively charged sialic acids leading to cell deformation and ultimately the activation of the EMT program [31, 35], leading to cell migration.

In addition, our study clarifies another misconception that most of the imported glucose in cancer is used toward the production of lactate and associated ATPs [36, 37], as the majority of imported glucose is allocated toward nucleotide synthesis across all cancer types.

It has long been recognized that high blood‐sugar level is a risk factor for cancer occurrence [38] but the detailed relationship between the two has been elusive. Our explanation is that an elevated level of blood sugar makes it easier to bring in sufficient levels of glucose to keep the cancer cells from being killed by alkalosis. This strongly suggests that lowering the blood sugar level could help to slow down the growth of cancer, knowing that cancer patients tend to have elevated levels of blood sugar (Figure S6). In a follow‐up study, we will conduct more quantitative analyses to estimate the average level of O$H^{-}$ production accurately among cells in cancer tissue as well as the rate of $H^{+}$ production by all the glucose‐based metabolisms as a function of the level of glucose import, dictated by the blood sugar level. Such analyses may provide highly useful guidance for controlling the level of blood sugar that may give the patients the best chance to survive.

Our study also helped to settle another issue that cancerous cell division is fundamentally different from the normal human‐cell division as the former is symmetric while the latter is asymmetric, knowing that (1) continuous cell division is part of the survival pathway for cells affected by FR, namely, cell will die if not being able to divide, and hence both daughter cells must be able to divide; and (2) stem cell division gives rise to one functional cell and one stem cell except for the first three generations of a fertilized egg cell.

## 4 CONCLUSION

This study presented strong evidence that it is the *de novo* synthesis rate of nucleotides that drives the cell‐division rate in cancer, not the other way around as it has long been believed. It is noteworthy that nucleotide synthesis, along with other acidifying metabolisms, is induced to produce $H^{+}$ions to keep the intracellular pH stable in cells affected by persistent intracellular FR; and driving persistent cell proliferation is just a side‐product of nucleotide synthesis, in order to have the synthesized nucleotides removed in a timely manner so this process can continue to produce $H^{+}$ions. The significance of this new insight lies in that it strongly suggests that limiting the uptake of glucose by cancer cells may become a highly effective way to treat cancer as cancer cells will die from alkalosis without a sufficient supply of glucose.

## 5 MATERIALS AND METHODS

### 5.1 Data preparation

We collected RNA‐seq data from 19 cancer types covering 6964 cancer samples and 3874 corresponding normal tissue samples from the TCGA (cancer samples) and GTEX (normal tissues) databases, respectively. These cancer types are selected in our study as they represent all the epithelial cell types with both sufficiently large numbers of cancer samples for each stage and of control normal samples.

Using standard processing procedures, the data were converted to transcripts per million (TPM) values and subsequently log_2_‐transformed. This preparation ensured data comparability across samples.

### 5.2 Major glucose metabolisms

We aim to estimate where the imported glucose goes and at what percentage in a specified cancer type. Based on our systematic analyses of all the sinks of glucose fluxes, we focus on eight glucose metabolic pathways, namely

1. Amino acids serine synthesis.
2. Ganglioside synthesis.
3. Glycosylation, including both O‐linked and N‐linked glycosylation.
4. Lactate production.
5. Lipid synthesis, covering triglycerides, sphingolipids, and unsaturated fatty acids.
6. Nucleotide *de novo* synthesis.
7. Sialic acid synthesis and deployment.
8. The first two steps of the tricarboxylic acid (TCA) cycle.

They represent the top glucose sinks and collectively cover at least 90% of the imported glucose across all the cancer types under study. Figure 2 shows the glucose metabolic pathways toward these sinks and the enzyme genes at the starting point of each of these pathways. These enzyme genes are determined to facilitate accurate estimate of glucose fluxes toward each of these major sinks.

### 5.3 Differential gene‐expression analyses

To identify statistically upregulated genes in each biological process of interest, we performed two‐sample *t*‐tests (unequal variance) on the log_2_‐transformed expression profiles. Specifically, for each gene:

1. We computed its fold change as the difference between the mean expressions across cancer versus control samples.
2. We then applied a multiple‐comparison correction using the Benjamini–Hochberg procedure [39] to control the false discovery rate.
3. Genes with a fold change ≥ 0 and adjusted *p*‐value < 0.05 were considered statistically upregulated. These genes in each biological process formed the basis for subsequent analyses.

### 5.4 Building a statistical model for glucose metabolisms

#### 5.4.1 Constructing features for each target metabolic process

For each cancer type, we construct a co‐expression network using all expressed genes from cancer samples in TCGA. Pairwise Pearson correlation coefficients are calculated among genes, and only gene‐pairs, represented as edges with an absolute correlation value exceeding 0.8 are retained. Within each glucose metabolic pathway, *hub* genes are identified based on degree centrality—specifically, genes whose degree centrality exceeds the mean value across all genes are selected. The expression levels of the hub genes are then averaged across samples to yield a single representative value for the pathway [40, 41]. Similarly, the “glucose uptake” process—defined by the expression of key transporter genes—is identified and averaged, and this value is used as the dependent variable in subsequent regression analyses aimed at quantifying how imported glucose is allocated among the different downstream pathways.

#### 5.4.2 Polynomial expansion and forward feature selection

We employ a polynomial feature expansion to allow for nonlinear relationships among the eight glucose metabolic pathways (predictors) and the glucose import (response). Specifically, given the original features $\left\{x_{1},x_{2},\ldots \}\right.$, a polynomial expansion up to degree 2 consists of all squared terms $\left(x_{i}^{2}\right)$ and pairwise interaction terms $\left(x_{i}x_{j}\right)$, making the predictor set $\phi_{k}\left(\vec{x}\right)$. To avoid overfitting and ensure parsimony, we apply forward feature selection:

1. Initialize with no feature.
2. Add iteratively one polynomial term at a time, which yields the largest improvement in $R^{2}$.
3. Stop when exceeding an $R^{2}$ threshold (set to 0.95) or reaching the maximum permissible features (to avoid *p*‐value invalidation when the feature count approaches the number of samples).

We then solve for {$\beta_{k}$} that fit an ordinary least squares model achieving the highest $R^{2}$:

(1) $$\text{glucose}\;\text{import}=\sum_{k=1}^{m}\beta_{k}\phi_{k}\left(\vec{x}\right).$$

#### 5.4.3 Model evaluation

We calculated the following:

1. $R^{2}$: The coefficient of determination, representing that the level of glucose uptake is explained by the selected model.
2. The overall *F*‐test *p*‐value: a measure of whether the regression model is significantly better than chance.

These metrics assess the fidelity of our polynomial regression model. By combining gene expression‐based feature construction and forward feature selection in the polynomial space, we capture potential nonlinearities among the major glucose metabolic pathways.

### 5.5 Two‐step FBA

To estimate glucose‐derived flux allocation across eight metabolic pathways in cancer tissues, we developed a two‐step flux balance analysis (FBA) [42] pipeline. In the first step, we leveraged transcriptomic data integrated with stoichiometric network constraints under pseudo‐steady‐state assumptions to derive cancer‐specific catalytic efficiency constants ($\frac{k_{\text{cat}}}{K_{m}}$), which subsequently informed flux capacity constraints. In the second step, we formulated flux estimation as a QP problem, combining linear flux‐balance objectives with quadratic penalties to ensure biologically plausible and balanced flux distributions.

#### 5.5.1 Metabolic network and internal nodes

We first constructed a glucose‐centric metabolic stoichiometric model based on pathway data from the KEGG database [18], as depicted in Figure 2. The network comprises the following major steps:

1. Glucose uptake: Mediated by transporters SLC2A1, SLC2A3, SLC5A1, SLC5A2, and SLC2A10, converting glucose to glucose‐6‐phosphate (G6P).
2. Pentose phosphate and glycolysis: G6P enters the pentose phosphate pathway (nucleotide synthesis) or is converted to fructose‐6‐phosphate (F6P). F6P is subsequently channeled either into UDP‐GlcNAc synthesis (precursor for sialic acid and glycosylation) or glycolytically converted to glyceraldehyde‐3‐phosphate (G3P).
3. Lipid synthesis and serine metabolism: G3P either forms triglycerides (lipids) or is metabolized into 3‐phosphoglycerate (3P), further converted to serine or pyruvate. Serine can remain as free serine or enter ceramide synthesis, subsequently fueling sphingolipid or ganglioside synthesis.
4. Pyruvate metabolism: Pyruvate either generates lactate or is converted to acetyl‐CoA, forming citrate to supply the TCA cycle or lipid synthesis.

Within this network, the metabolites G6P, F6P, UDP‐GlcNAc, G3P, 3P, serine, ceramide, pyruvate, acetyl‐CoA, and citrate serve as internal nodes (transit metabolites). The terminal nodes—nucleotide synthesis, sialic acid, glycosylation, lipid forms, lactate, TCA intermediates, gangliosides, and remaining serine—act as sink nodes (Figure 2).

#### 5.5.2 Estimation and validation of cancer‐specific $\frac{k_{\text{cat}}}{K_{m}}$ values

1. Stoichiometric network reconstruction: For each of the above pathways, KEGG KGML files were parsed to obtain reaction identifiers, balanced reaction stoichiometries, EC numbers, and gene–protein–reaction (GPR) mappings, resulting in a genome‐scale stoichiometric matrix (S).
2. Construction of the enzyme activity proxy matrix (E) by gene weighting: To construct a proxy for enzyme activity that is context‐specific to each cancer type, we implemented a gene‐weighting strategy informed by both expression magnitude and statistical relevance. Initially, differential expression (DE) analyses were performed on $\log_{2}$‐transformed gene‐expression data, comparing cancer samples to their corresponding normal tissue controls. This analysis yields for each gene, a $\log_{2}$ fold‐change ($\log_{2}$ FC) and a Benjamini–Hochberg‐adjusted *p*‐value ($p_{\text{adj}}$) [39].
  For each gene g, a composite weight $w_{g}$ was calculated as the product of a fold‐change factor ($w_{\text{FC}}$) and a significance factor ($w_{\text{FDR}}$):
(2) $$w_{\text{FC}}=2^{\gamma \cdot \log_{2}\text{FC}},$$
  where γ=0.75 is an exponent to temper the influence of extreme FCs, and
(3) $$w_{\text{FDR}}={\left(1-p_{\text{adj}}\right)}^{\eta },$$
  with η=0.7 penalizing genes with low statistical significance.
  The enzyme activity proxy for reaction j in sample i, denoted as $E_{ij}$, was computed as:
(4) $$E_{ij}=\sum_{g\in R_{j}}w_{g}\cdot {\text{expression}}_{ig},$$
  where $R_{j}$ denotes the set of genes encoding the enzymes catalyzing reaction j, and ${\text{expression}}_{ig}$ is the TPM expression of gene g in sample i. This formulation ensures that each enzyme contributes to the total activity proxy in proportion to both its expression and its statistical association with the cancer type. The resulting E matrix was further normalized by its median value to ensure numerical stability in downstream optimization.
3. NNLS for $\frac{k_{\text{cat}}}{K_{m}}$ estimation: The flux through each reaction ($v_{ij}$) was modeled as proportional to the product of the unknown catalytic efficiency (${\text{eff}}_{j}=\frac{k_{\text{cat},j}}{K_{m,j}}$) and the enzyme activity proxy ($E_{ij}$):
(5) $$v_{ij}={\text{eff}}_{j}\cdot E_{ij}.$$
  Under the pseudo steady‐state assumption, fluxes must satisfy the mass‐balance constraint:
(6) S·v=0.
  To infer the vector of catalytic efficiencies (eff), we formulated a regularized nonnegative least squares (NNLS) optimization problem:
(7) $$\min_{\text{eff}\geq 0}∥S\cdot \left(\text{eff}\circ E\right){∥}_{2}^{2}+\sum_{j\in \text{unknowns}}\lambda_{j}∥{\text{eff}}_{j}-{\text{eff}}_{j}^{\left(0\right)}{∥}_{2}^{2},$$
  where ${\text{eff}}^{\left(0\right)}$ represents prior estimates for $\frac{k_{\text{cat}}}{K_{m}}$, derived from literature‐curated values in the BRENDA [22] database and predictions from the CatPred deep learning model [23]. For reactions lacking experimental or predicted values, the global median of all prior efficiencies was used as a fallback. The regularization terms ($\lambda_{j}$) control the strength of prior adherence, preventing overfitting in the presence of limited or noisy data.
4. Validation of $\frac{k_{\text{cat}}}{K_{m}}$ by EC classification: To validate the biological relevance of our estimated $\frac{k_{\text{cat}}}{K_{m}}$ values, we mapped each reaction to its primary EC classification. Differences in median $\frac{k_{\text{cat}}}{K_{m}}$ values across EC classes were statistically tested using the Kruskal–Wallis *H*‐test (significance threshold *p* < 0.05). Distributions were visualized using combined boxplots on a logarithmic scale to clearly illustrate variability and central tendencies.

#### 5.5.3 Flux estimation via QP

Utilizing the previously estimated $\frac{k_{\text{cat}}}{K_{m}}$ values, we formulated the estimation of glucose‐derived fluxes into eight metabolic pathways as a QP problem. This formulation integrates linear programming (LP)‐based flux constraints with a quadratic penalty to avoid biologically unrealistic “winner‐take‐all” flux distributions.

##### 5.5.3.1 Variables and constraints

1. Glucose uptake ($v_{\text{glc}\_\text{in}}$): Represents the total influx of glucose, mediated by specified glucose transporters.
2. Mass‐balance constraints: For each internal metabolite m:
(8) $$\sum_{r\in \text{influx}\left(m\right)}v_{r}-\sum_{r\in \text{efflux}\left(m\right)}v_{r}=0.$$
3. Sink reactions: Terminal pathways (e.g., nucleotide synthesis, lactate, TCA intermediates, and ganglioside synthesis) are represented by sink variables $v_{\text{sink}}$. These collectively consume glucose‐derived carbon exiting the internal metabolite nodes.
4. Flux balance constraint: Total influx to sinks equals total glucose uptake:
(9) $$\sum_{\text{sink}}v_{\text{sink}}=v_{\text{glc}\_\text{in}},$$
  (minor metabolic sinks, such as hyaluronic acid synthesis, were assumed to be negligible in this analysis for simplicity).
5. Flux capacity constraints (upper bounds): For each sink reaction, an upper bound ($B_{\text{sink}}$) was defined using a combination of the pathway activity score $G_{\text{sink}}$ (computed via ssGSEA, and transformed via an exponential function to yield a positive scaled pathway activity score) and the efficiency rate constant ($\frac{k_{\text{cat}}}{K_{m}}$):
(10) $$B_{\text{sink}}=G_{\text{sink}}\times \frac{k_{\text{cat}}}{K_{m}}.$$
  Thus, each flux variable satisfies:
(11) $$0\leq v_{\text{sink}}\leq B_{\text{sink}}.$$

##### 5.5.3.2 Objective function (balanced quadratic formulation)

To avoid unrealistic flux distributions, we defined an objective function combining linear pathway weights and a quadratic penalty:

(12) $$\max_{v_{\text{sink}}}\sum_{\text{sink}}\left(w_{\text{sink}}\cdot v_{\text{sink}}-\lambda \cdot v_{\text{sink}}^{2}\right),$$

where $w_{\text{sink}}$ are weights derived by applying a square‐root normalization of the expression‐based bounds $B_{\text{sink}}$:

(13) $$w_{\text{sink}}=\frac{\sqrt{B_{\text{sink}}}}{\sum_{{\text{sink}}^{\prime }}\sqrt{B_{{\text{sink}}^{\prime }}}},$$

and $\lambda \cdot v_{\text{sink}}^{2}$ serves as a quadratic penalty to penalize disproportionally large fluxes, promoting balanced and biologically realistic flux solutions.

This QP formulation—integrating mass balance, catalytic constraints, and activity‐based weighting—ensures accurate modeling of metabolic flux distributions, balancing theoretical rigor with biological realism.

### 5.6 Further modeling of lactate fluxes

The flux directed toward lactate production ($v_{\text{lactate}}$), as determined by the primary FBA, constitutes a critical metabolic branch point. To further elucidate the fate of this flux, we implemented a secondary QP model designed to optimally partition the lactate flux between extracellular export and intracellular utilization via protein lactylation.

#### 5.6.1 Derivation of pathway‐specific weights

To inform the objective function of the QP model, pathway‐specific activity scores were calculated to represent the relative activities of lactate export and lactylation. Two distinct gene sets were curated: one corresponding to lactate efflux transporters and the other to enzymes involved in protein lactylation. For each gene set, a pathway activity score was computed using the ssGSEA method.

After computing the ssGSEA scores ($s_{\text{export}}$ and $s_{\text{lactylation}}$) for these pathways, the raw activity scores were transformed into robust weights as follows:

1. Logarithmic compression: Each score is compressed to manage a wide dynamic range using the natural logarithm:
(14) $$z_{i}=\ln\left(1+s_{i}\right),$$
  where i∈{export,lactylatio}.
2. Temperature‐scaled softmax: The compressed values are converted into a softmax distribution to yield preliminary weights:
(15) $${\tilde{w}}_{i}=\frac{\exp\left(z_{i}/\tau \right)}{\exp\left(z_{\text{export}}/\tau \right)+\exp\left(z_{\text{lactylation}}/\tau \right)},$$
  where τ>0 is the temperature parameter controlling the sharpness of the weight distribution.
3. Blending with uniform prior: To prevent extreme or degenerate solutions, the softmax weights are blended with a uniform prior (α):
(16) $$w_{i}=\alpha \cdot 0.5+\left(1-\alpha \right)\cdot {\tilde{w}}_{i},$$
  where 0<α<1 determines the strength of the prior.

The resulting weights, $w_{\text{export}}$ and $w_{\text{lactylation}}$, serve as the linear coefficients in the QP objective function.

#### 5.6.2 Lactate flux partition model

The partitioning of lactate flux was formulated as a QP problem, maximizing a weighted sum of export and lactylation fluxes, subject to mass conservation. The optimization problem is defined as:

(17) $$\max_{v_{\text{export}},v_{\text{lactylation}}}w_{\text{export}}\;v_{\text{export}}+w_{\text{lactylation}}\;v_{\text{lactylation}}-\lambda \left(v_{\text{export}}^{2}+v_{\text{lactylation}}^{2}\right)\text{subject}\;\text{to}:v_{\text{export}}+v_{\text{lactylation}}=v_{\text{lactate}}\;v_{\text{export}}\geq 0,v_{\text{lactylation}}\geq 0.$$

Here, the linear terms (w·v) drive the solution toward pathways with higher inferred activities, whereas the quadratic penalty term ($-\lambda \cdot v^{2}$) models encourages a balanced allocation between fates. The mass balance constraint ensures that all produced lactate is assigned to either export or lactylation. By solving this QP, we obtain a biologically interpretable and quantitative estimate of how cancer cells balance lactate efflux and internal utilization through protein lactylation.

### 5.7 Estimation of pathway‐specific proton (H^+^) production

Following the determination of pathway‐specific metabolic fluxes across the glucose metabolic network, we quantified the net proton (H^+^) production to evaluate the implications of metabolic reprogramming on intracellular acidification. To achieve this, we defined a pathway‐specific proton production factor ($h_{i}$) derived from the enzymatic reactions annotated in the KEGG database [18]. Specifically, for each reaction associated with pathway i, the net stoichiometric coefficient of protons (KEGG ID: C00080) was calculated based on its balanced chemical equation. The proton production factor for pathway i, denoted $h_{i}$, was computed as the average of these reaction‐specific proton coefficients:

(18) $$h_{i}=\frac{1}{\left|R_{i}\right|}\sum_{r\in R_{i}}h_{r},$$

where $R_{i}$ denotes the set of enzymatic reactions in pathway i, and $h_{r}$ represents the net proton stoichiometric coefficient for reaction r.

The total proton production flux ($J_{H^{+},i}$) for each pathway was then obtained by multiplying the corresponding pathway flux ($v_{i}$), estimated via QP (Section 5.5.3.2), by the calculated proton factor ($h_{i}$):

(19) $$J_{H^{+},i}=v_{i}\cdot h_{i}.$$

This formulation enabled a quantitative assessment of proton dynamics associated with each glucose‐derived metabolic pathway, thereby facilitating insights into the potential contribution of metabolic shifts to cellular acidification in cancer.

### 5.8 Causal inference on the role of nucleotide synthesis in cell cycle progression

Following the estimation of metabolic fluxes, we sought to determine whether the activation of the nucleotide synthesis pathway exerts a causal influence on cell cycle progression. A central analytical challenge in addressing this question is in distinguishing the direct impact of nucleotide synthesis from the confounding effects of cellular proliferation, which simultaneously influences both nucleotide metabolism and cell division. To rigorously address this, we implemented a formal causal inference method grounded in Judea Pearl’s SCM framework [43].

#### 5.8.1 Definition of causal variables

We constructed a causal model based on three key variables extracted from the tumor transcriptomic data, which are defined as follows:

1. Outcome (Y): cell cycle activity: The outcome was quantified as the normalized $\log_{2}$‐transformed expression level of *MKI67*, a canonical and well‐established molecular marker [44] for cell proliferation.
2. Treatment (T): nucleotide synthesis pathway activity: To specifically capture the activity level of the nucleotide synthesis pathway in cancers, we employed a two‐step procedure:
  - A DE analysis was performed between cancer and normal tissue samples to identify genes in the nucleotide synthesis pathway that exhibited significant upregulation in cancer samples (Benjamini–Hochberg adjusted *p*‐value < 0.05). This set of genes represents cancer‐specific activation signature of the nucleotide synthesis pathway.
  - The ssGSEA scores were computed for each cancer sample using this refined gene set. The resulting enrichment score served as a quantitative measure of the treatment variable, T.
3. Confounder (W): cellular proliferation signature: To explicitly control for overall cell‐proliferation activities that may confound the direct relationship between nucleotide synthesis and cell cycle progression, we defined an independent cell‐proliferation signature as follows:
  - A separate DE analysis contrasted highly proliferative normal human tissues (obtained from external public datasets: GSE201955, GSE111085, and GSE171012) against quiescent normal tissues (GTEx). The top 500 most significantly upregulated genes formed a general cell‐proliferation signature.
  - To ensure statistical independence and specificity, we removed genes that are present in both this cell‐proliferation signature and the nucleotide synthesis signature. The resulting signature was then employed in a separate ssGSEA analysis of cancer samples to generate the confounder variable, W.

Prior to inclusion in the causal model, all three variables (T, Y, and W) were standardized (*z*‐score normalized) across all tissue samples.

#### 5.8.2 Causal model specification and estimation

A directed causal graph was constructed in which the confounder (general proliferation, W) simultaneously influences both the treatment (nucleotide synthesis, T) and the outcome (cell cycle activity, Y). This specification reflects our hypothesis of W as a common cause confounding the direct relationship between T and Y.

The causal effect of nucleotide synthesis activity on cell cycle progression was estimated using Pearl’s backdoor adjustment criterion [43]. The average causal effect is formally identified via the following adjustment formula:

(20) $$P\left(Y|\text{do}\left(T\right)\right)={\text{∫}}_{W}P\left(Y|T,W\right)P\left(W\right)dW.$$

The backdoor adjustment is implemented through a multivariable linear regression model:

(21) $$E\left[Y\right]=\beta_{0}+\beta_{\text{ATE}}\cdot T+\beta_{W}\cdot W+\epsilon .$$

In this formulation, the estimated regression coefficient $\beta_{\text{ATE}}$ directly corresponds to the ATE [45] of the nucleotide synthesis pathway activity on cell cycle activity, explicitly controlling for the confounding influence of the general cellular proliferation process.

#### 5.8.3 Model validation and sensitivity analysis

To evaluate the significance and stability of the causal estimate, we conducted two complementary validation analyses:

1. Permutation test: To empirically assess the probability of obtaining the observed causal effect by random chance, we performed a permutation test (N=500). In each permutation, the treatment variable T was randomly reshuffled across samples to disrupt its association with the outcome Y. Then the ATE was recalculated in each permuted dataset, obtaining an empirical null distribution. The resulting permutation *p*‐value was computed as the proportion of permuted absolute ATEs that were greater than or equal to the original observed absolute ATE.
2. Refutation test (unobserved confounder simulation): To evaluate sensitivity to potential unmeasured confounders, a refutation test was performed by introducing a simulated latent variable hypothesized to influence both T and Y. By systematically varying the simulated confounder’s strength, we assessed how substantially the original ATE estimate could change. This analysis provided insights into the robustness of our conclusions against hidden biases and unobserved confounding influences.

Finally, to visually present the causal relationship between the nucleotide synthesis activity and cell cycle progression, we generated a partial regression plot, which graphically illustrates the direct association between the treatment T and the outcome Y after statistically removing the linear effects of the confounder W.

### 5.9 Regression analysis for rate estimation

To quantify how specific metabolic programs relate to downstream phenotypes, we used a two‐step framework that combines supervised dimensionality reduction with linear regression. We applied this framework to three questions: (1) how nucleotide‐synthesis activity relates to cell proliferation, (2) how sialic‐acid deployment relates to cell migration, and (3) how the eight glucose‐downstream pathways jointly explain the cytosolic Fenton reaction.

#### 5.9.1 Dimensionality reduction via PLS

Given a high‐dimensional predictor matrix $Χ\in R^{n\times p}$ (rows: tumor samples; columns: genes from a prespecified set) and an outcome vector $Y\in R^{n\times 1}$, we apply PLS regression [46] to extract a small number of latent components. Unlike principle component analysis, which maximizes variance in Χ alone, PLS maximizes the covariance between Χ and Y. Formally, PLS finds

(22) $$Χ\approx TP^{T}\;\text{and}\;Y\approx TQ,$$

where $T\in R^{n\times k}$ contains the PLS scores (“signatures”), P are loadings for Χ, and Q links T to Y. In the proliferation and migration analyses, Χ comprises nucleotide‐synthesis or sialic‐acid genes, respectively, and Y is either $\log_{2}$‐transformed MKI67 expression (proliferation) or the mean $\log_{2}$‐expression of migration‐related genes (migration).

For the Fenton reaction analysis, we applied the same procedure. Let ${Χ}_{F}\in R^{n\times p_{F}}$ be the expression matrix of the curated Fenton reaction gene set and $y_{F}$ the per‐sample mean expression of those genes. A one‐component PLS (PLS1) fit to $\left({Χ}_{F},y_{F}\right)$ yields the Fenton reaction score, defined as the first *Χ*_*F*_‐score ${t_{F}=Χ}_{F}w_{F}$. For each glucose‐downstream pathway k (eight in total) with gene set $G_{k}$, we analogously construct ${Χ}_{k}$ and $y_{k}$, fit PLS1, and take the first *Χ*_*K*_‐score $t_{k}$ as that pathway’s score. All scores are centered by the PLS routine and carried forward to regression.

#### 5.9.2 Multiple linear regression

The PLS scores $T\in R^{n\times k}$ serve as predictors in an ordinary least‐squares model,

(23) $$Y_{i}=\beta_{0}+\sum_{j=1}^{k}\beta_{j}{Τ}_{ij}+\epsilon_{i},\epsilon_{i}∼N\left(0,\sigma^{2}\right),$$

Model fit is summarized by the coefficient of determination

(24) $$R^{2}=1-\frac{\sum_{i=1}^{n}{\left(Y_{i}-{\hat{Y}}_{i}\right)}^{2}}{\sum_{i=1}^{n}{\left(Y_{i}-\bar{Y}\right)}^{2}}.$$

We applied this same specification to three analyses, differing only in the definition of Y and the predictor matrix Τ (with k as the number of retained PLS components—one in our implementation unless stated otherwise):

1. Proliferation (nucleotide synthesis): Y is $\log_{2}$‐transformed MKI67 expression; Τ comprises the nucleotide‐synthesis PLS scores for each sample. Here, $R^{2}$ quantifies how much of the variance in proliferation is explained by the nucleotide‐synthesis signature.
2. Migration (sialic‐acid deployment): Y is the mean $\log_{2}$‐expression of a curated migration gene set; Τ comprises the sialic‐acid deployment PLS scores. The resulting $R^{2}$ reflects the explanatory power of the sialic signature for migration.
3. Fenton reaction explained by glucose pathways: ${Y=t}_{F}$ is the Fenton reaction score (first PLS *Χ*‐score from the Fenton gene set); $Τ=\left[t_{1},\ldots ,t_{8}\right]$ are the eight glucose‐pathway scores (each a first PLS *Χ*‐score from the corresponding pathway gene set). The fitted value ${{\hat{t}}_{F}=\beta }_{0}+\sum_{k=1}^{8}\beta_{k}t_{k}$ is the predicted Fenton reaction score, and the associated $R^{2}$ measures the fraction of variance in the Fenton reaction explained jointly by the eight pathways.

In all cases, a higher $R^{2}$ indicates that the PLS‐derived signatures account for a larger share of variability in the outcome.

### 5.10 Cell‐based experimental validation

#### 5.10.1 Cell culture

The HGC‐27 and AGS gastric cancer cell lines, along with the GES‐1 normal gastric mucosal epithelial cell line, were procured from Procell and cultured in RPMI‐1640 medium (Vivacell) supplemented with 10% fetal bovine serum (FBS, Moregate) and 1% penicillin‐streptomycin antibiotic (Procell). The THLE‐2 immortalized normal hepatocyte cell line was obtained from Procell and maintained in THLE‐2 Cell Complete Medium (Procell). The LM3 and Huh7 hepatocellular carcinoma cell lines were sourced from the BeNa Culture Collection and cultured in high‐glucose Dulbecco’s modified eagle medium (DMEM, Vivacell) supplemented with 15% and 10% FBS, respectively, along with 1% penicillin‐streptomycin. Low‐glucose DMEM and glucose‐free RPMI‐1640 medium were obtained from Vivacell and Procell, respectively. All cell lines were cultured in a humidified incubator at 37°C with 5% CO_2_ and were subjected to testing and authentication to confirm their identity and purity.

#### 5.10.2 Induction of FR in cultured cells

Sustained FR were induced in the cytosol by adding FeSO_4_ (Sigma, F8633), H_2_O_2_ (Aladdin, 7722‐84‐1), and L‐ascorbic acid (Sigma, A4544) to the culture medium.

#### 5.10.3 Reverse transcription quantitative PCR (RT‐qPCR)

Total mRNA was isolated from Huh7 cells using the RNA‐easy isolation reagent (R701‐01, Vazyme) following the manufacturer’s instructions. The extracted RNA was resuspended in RNase‐free H_2_O and reverse‐transcribed into cDNA using the HiScript II 1st Strand cDNA Synthesis Kit with gDNA wiper (R212‐02, Vazyme). Real‐time quantitative PCR analysis targeted the genes *TYMS* (forward strand: ATCCAACACATCCTCCGCTG; reverse strand: ACACCCTTCCAGAACACACG), *RRM2* (forward: GCCACACCATGAATTGTCCG; reverse: ATGGTAAGTCACAGCCAGCC), and *NME1* (forward: TACCATCCCCGACCATCTGA; reverse: TCTATTAGGTCAGGTTATTCAGTGT) using the Taq Pro Universal SYBR qPCR Master Mix (Q712‐02, Vazyme). Relative gene expression levels were calculated using the 2^−^ΔΔCT method [47], with GAPDH serving as the internal reference gene.

#### 5.10.4 Live cell detection

A working solution of calcein acetoxymethyl ester (AM) and propidium iodide (PI) was prepared by combining calcein AM and PI solutions from the Calcein/PI Cell Viability/Cytotoxicity Assay Kit (C2015, Beyotime). Cells were incubated with this working solution at 37°C in the dark for 30 min. Following incubation, staining was visualized using a fluorescence microscope.

#### 5.10.5 Statistical analysis

All experiments were performed with a minimum of three biological replicates per experimental group. Statistical analyses were conducted using GraphPad Prism software. Comparisons between group means were performed using Tukey’s test following two‐way analysis of variance. Data are presented as mean ± standard deviation. Statistical significance was defined as *p*‐value < 0.05, with levels denoted as *p‐value < 0.05, **p‐value < 0.01, ***p‐value < 0.001, and ****p‐value < 0.0001.

## AUTHOR CONTRIBUTIONS

**Xuechen Mu**: Conceptualization; data curation; formal analysis; methodology; validation; visualization; writing—original draft; writing—review and editing. **Aoran Liu**: Investigation; validation. **Rui Shi**: Conceptualization; formal analysis; methodology. **Long Xu**: Conceptualization; data curation; methodology; visualization. **Zhenyu Huang**: Data curation; formal analysis; methodology. **Ye Zhang**: Data curation; investigation; validation. **Ying Xu**: Conceptualization; formal analysis; funding acquisition; methodology; supervision; writing—original draft; writing—review and editing.

## CONFLICT OF INTEREST STATEMENT

Ying Xu is the Editorial Board Member of *Quantitative Biology*. He was excluded from the peer‐review process and all editorial decisions related to the acceptance and publication of this article. Peer review was handled independently by the other editors to minimize bias. The authors declare no conflicts of interest.

## ETHICS STATEMENT

This study used only publicly available, de‐identified data and involved no new experiments on humans or animals. Accordingly, institutional review board approval and informed consent were not required.

## Supporting information

Supporting Information S1

## Data Availability

All datasets analyzed in this study are publicly available from the following repositories: The Cancer Genome Atlas (TCGA) via the GDC portal (portal.gdc.cancer.gov), GTEx (gtexportal.org), NCBI GEO (ncbi.nlm.nih.gov/geo), KEGG (kegg.jp), and BRENDA (brenda‐enzymes.org). The analysis code will be made available on GitHub (Mxc666/GLUCOSE‐METABOLISM).
