# Supplementary material for: On why cancer cells require a great amount of glucose
Source: Quant Biol. 2025 Dec 5;14(2):e70025. doi: 10.1002/qub2.70025 (PMC12806031; doi:10.1002/qub2.70025)
Supplement: Supplementary file 1 — Supporting Information S1 [file QUB2-14-e70025-s001.docx]

**SUPPLEMENTARY MATERISAL**

**
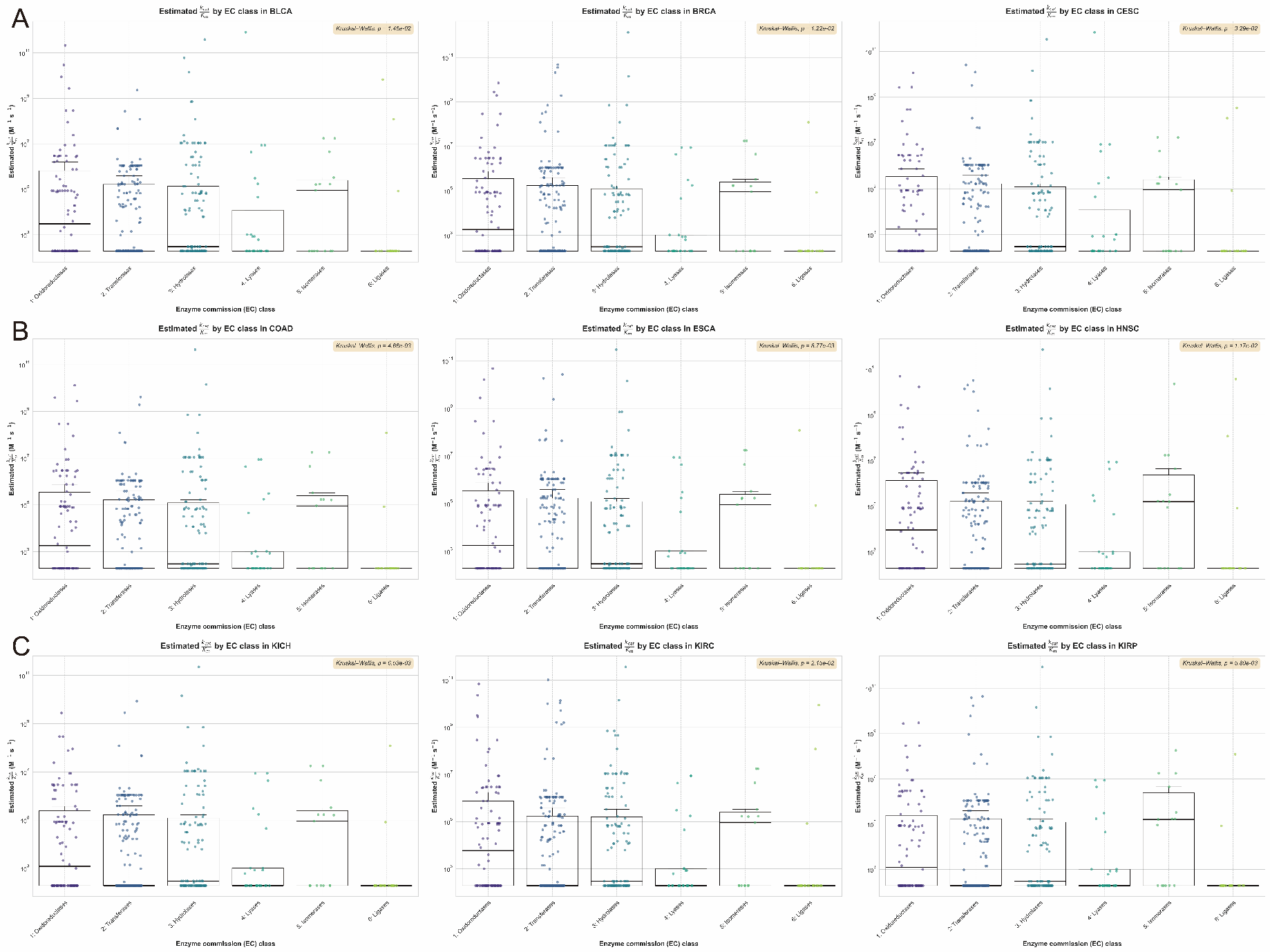
**

**
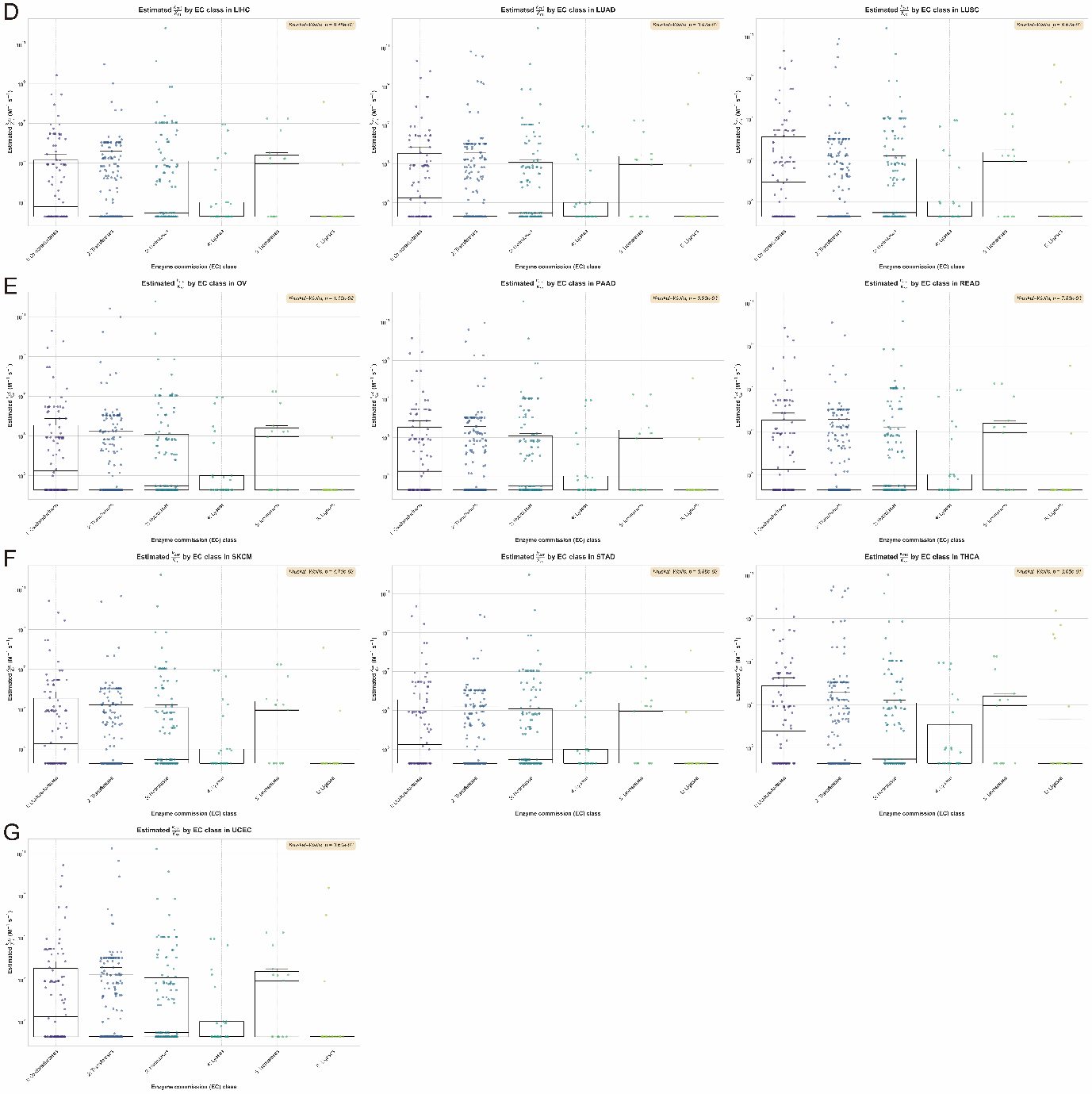
**

**Figure S1.** Validation of estimated catalytic efficiency constants ($\frac{k_{cat}}{K_{m}}$) across 19 cancer types based on Enzyme Commission (EC) classification. Each panel displays boxplots of $\frac{k_{cat}}{K_{m}}$ estimates across EC classes (1: Oxidoreductases, 2: Transferases, 3: Hydrolases, 4: Lyases, 5: Isomerases, 6: Ligases), with black line indicating median values and statistical significance assessed via Kruskal-Wallis H-test.

**
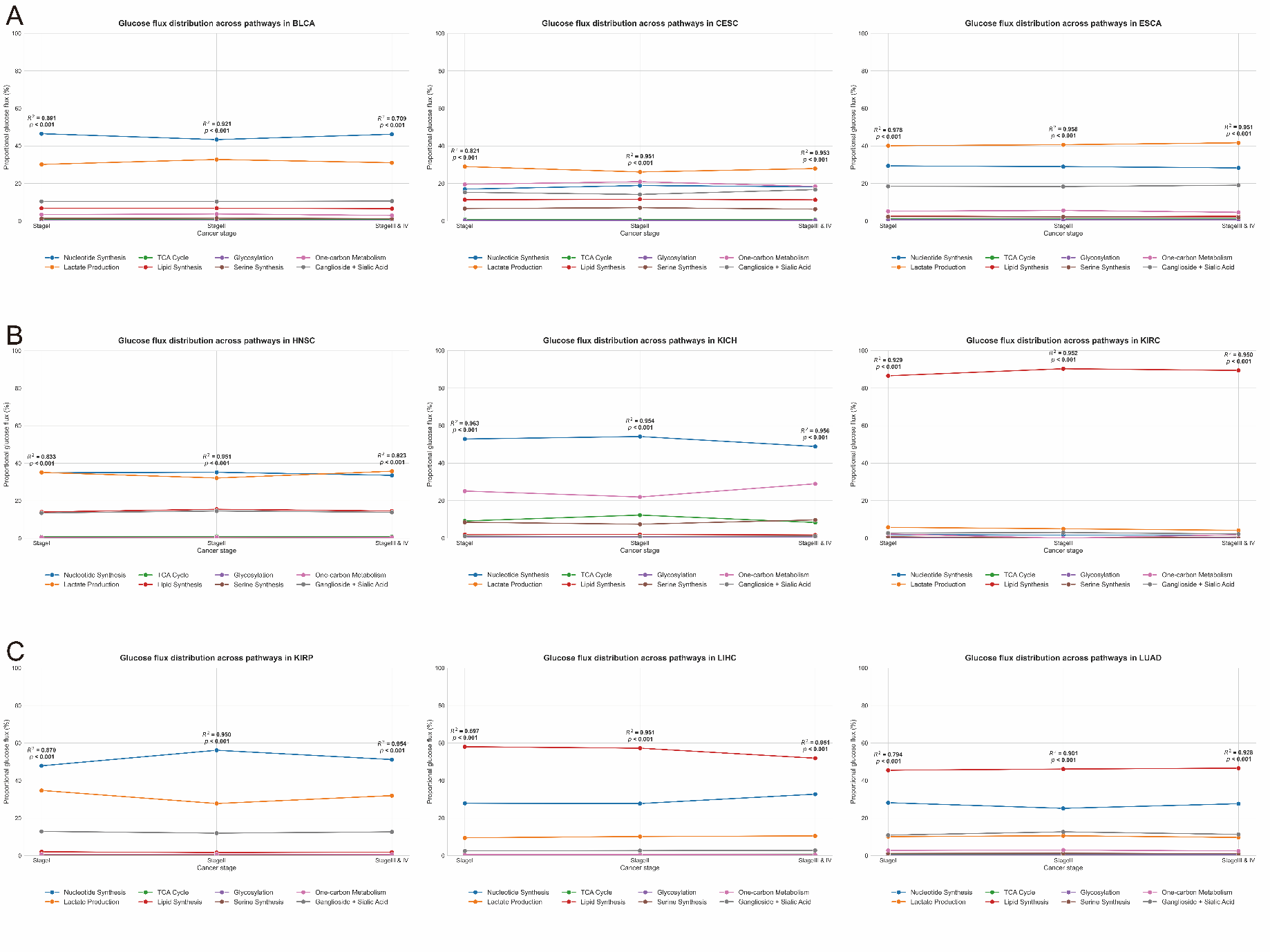
**


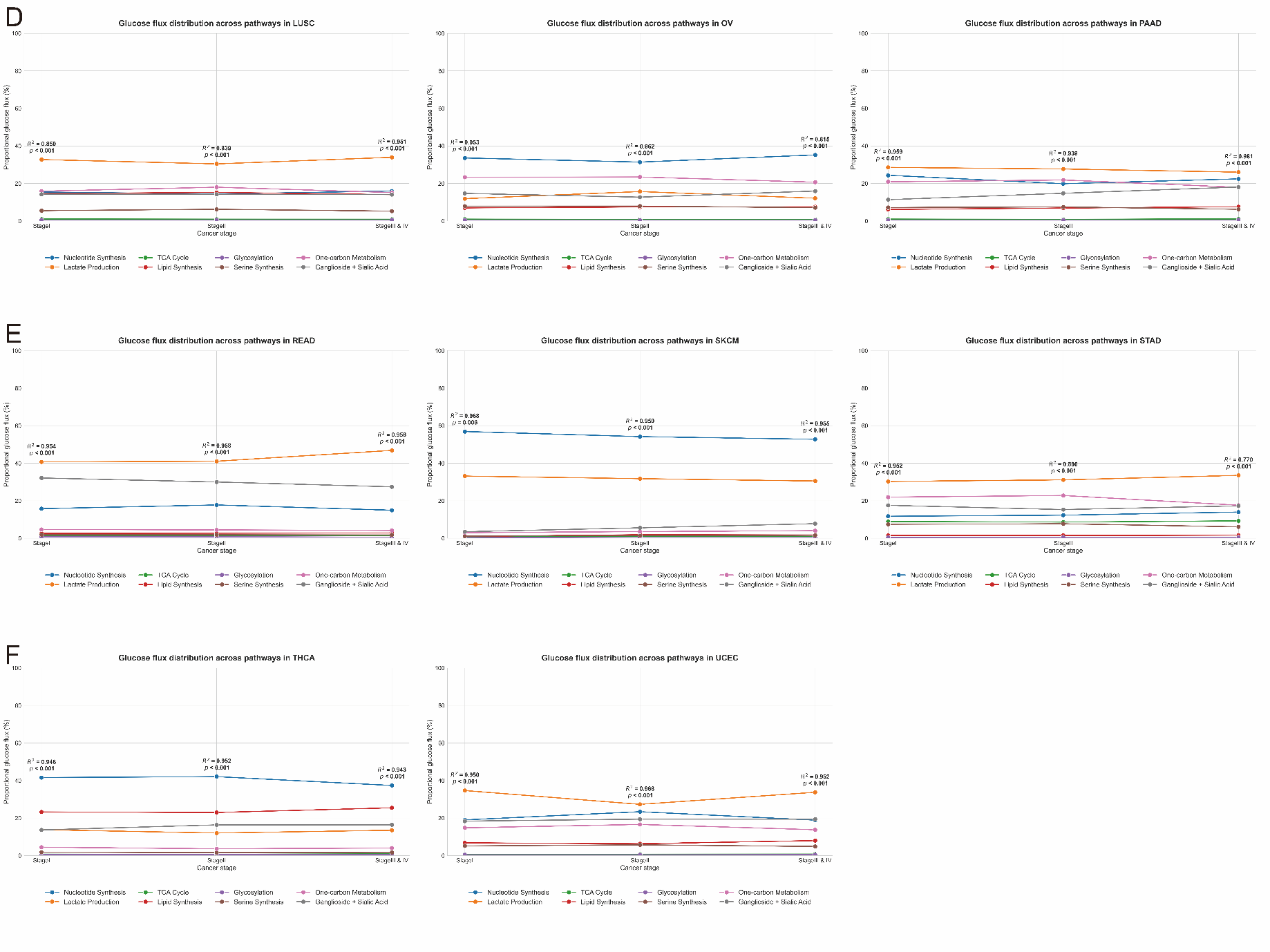


**Figure S2**: Glucose allocation in 17 cancer types. Each panel shows the percentage of glucose import allocated to eight glucose metabolic pathways across cancer stages, respectively.

**
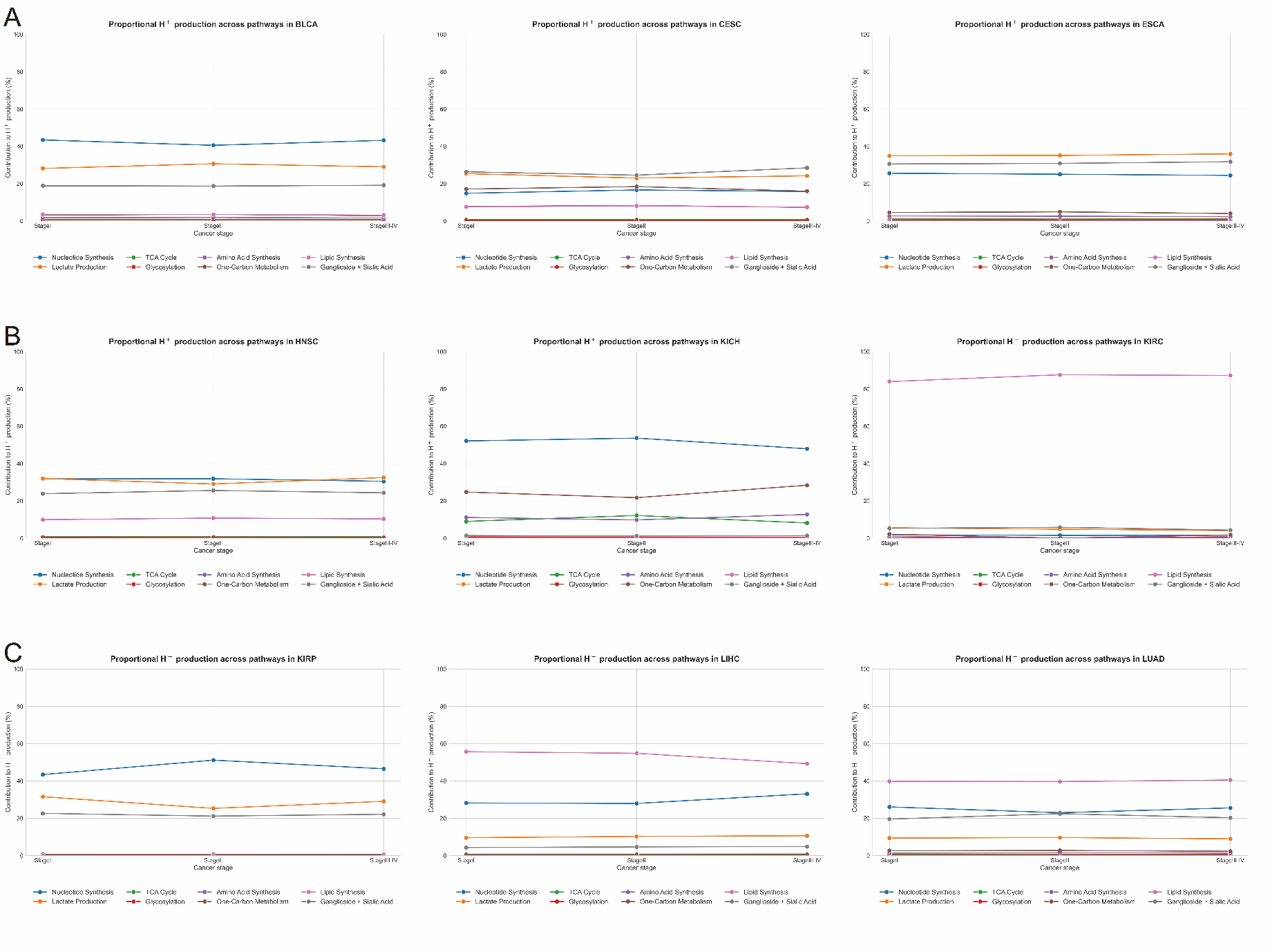
**


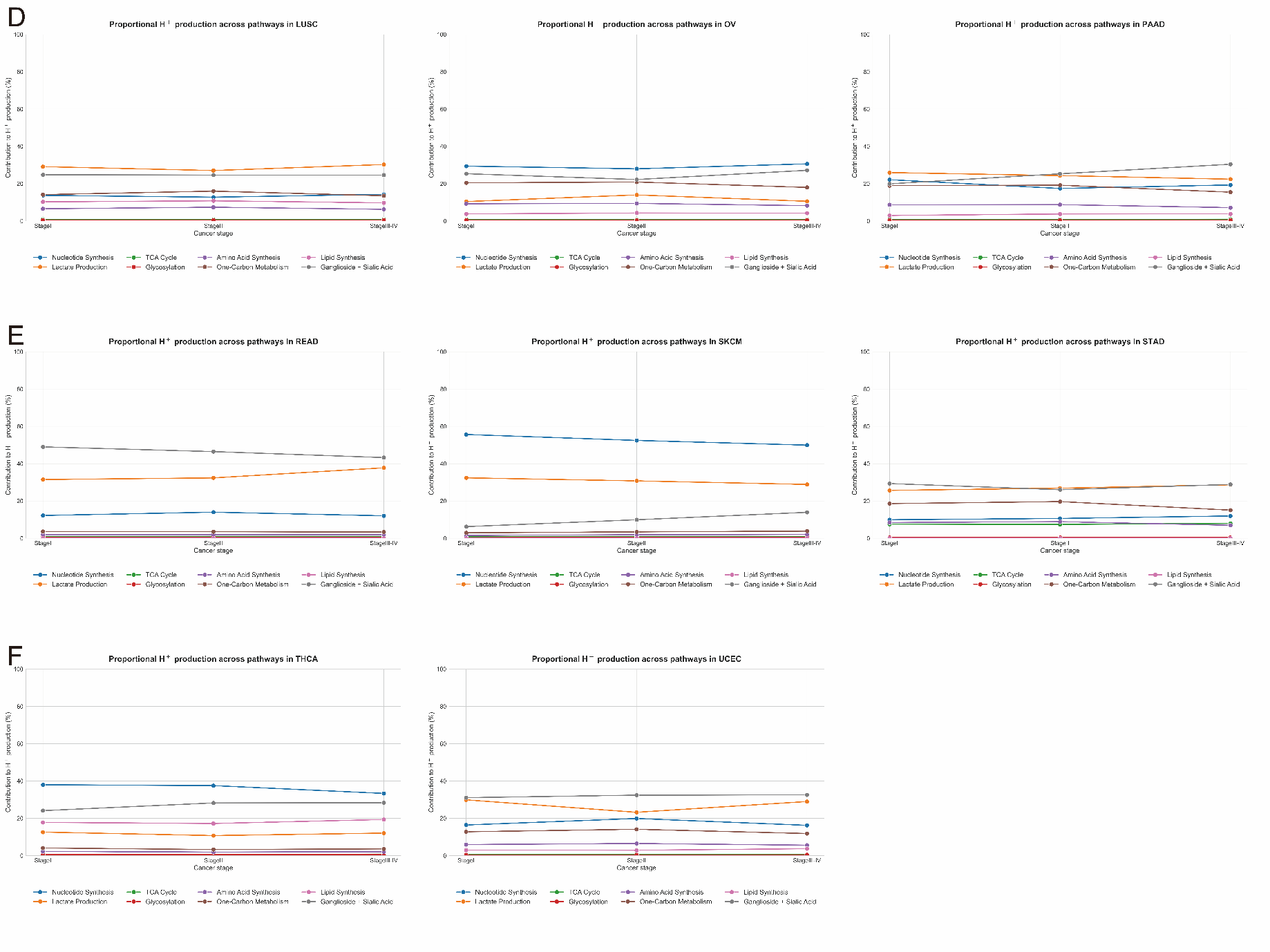


**Figure S3:** Percentage of H^+^ production by each glucose metabolic pathway in 17 cancer types. Each panel displays the percentage of $H^{+}$ production in each cancer type.

**
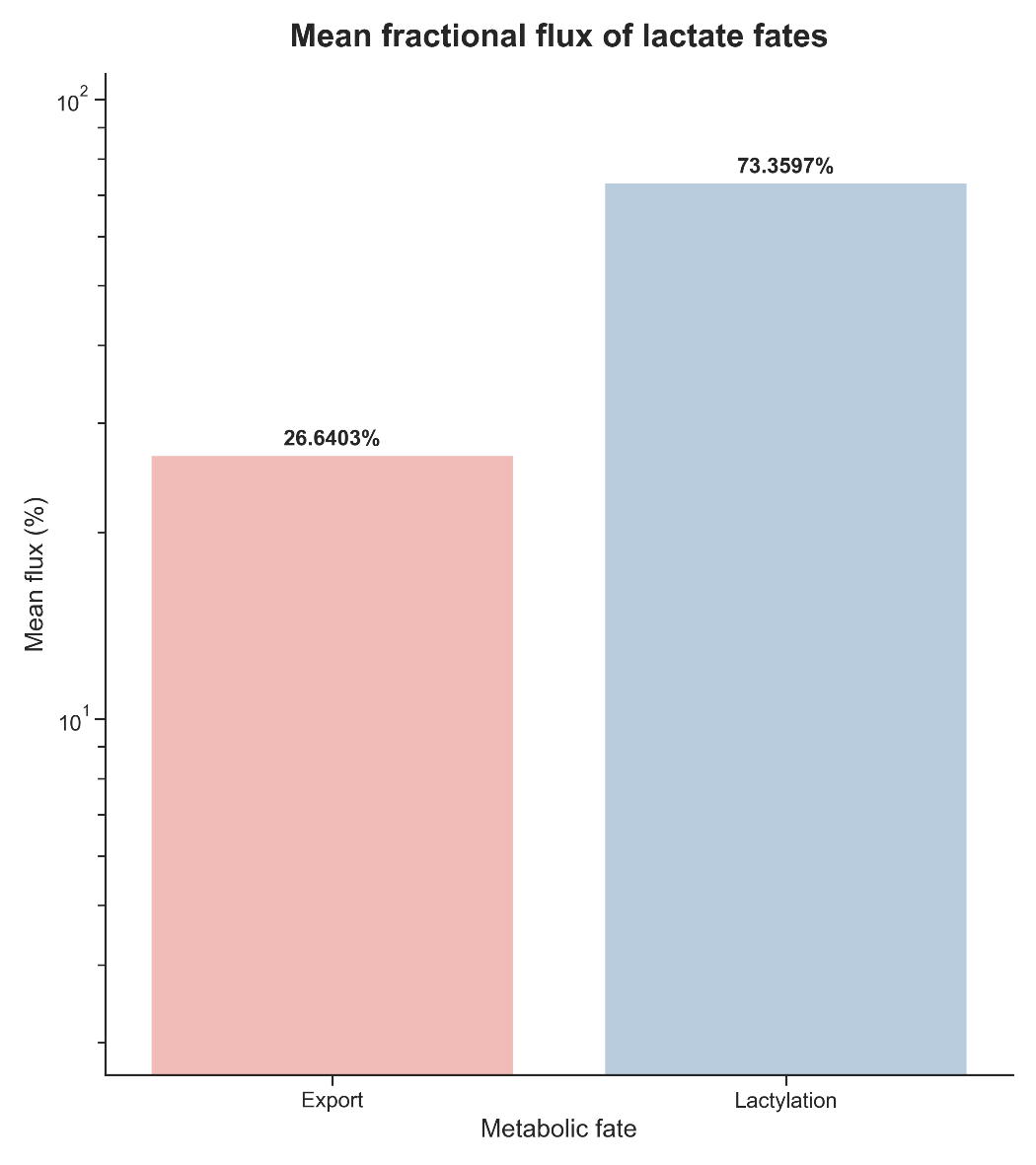
**

**Figure S4:** Metabolic flux distribution of lactate production across 19 cancer types. Bar graph illustrating the mean percentage of lactate flux allocated to extracellular export *versus* intracellular protein lactylation across 19 cancer types, derived from metabolic flux modeling of glucose metabolism.

**
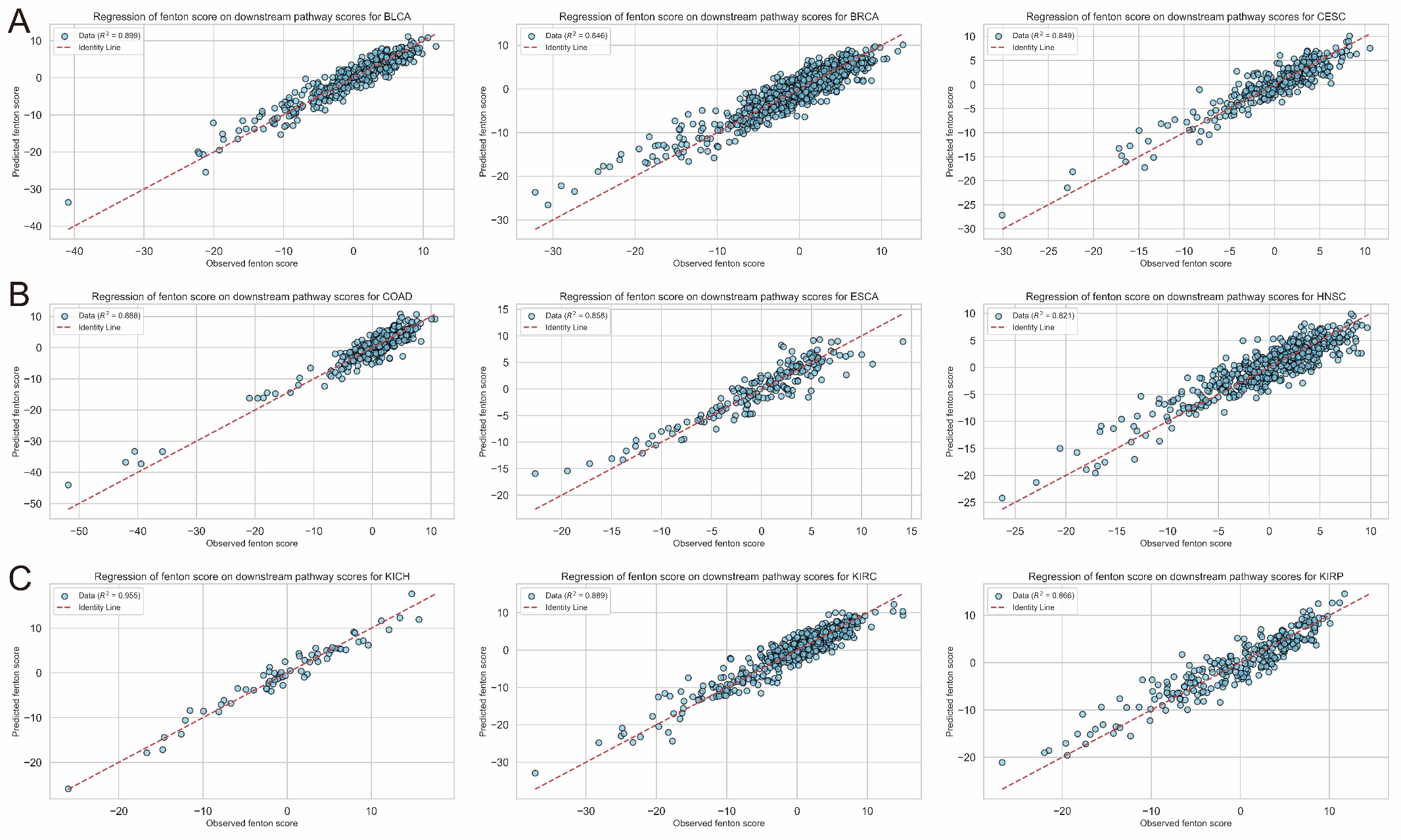
**

**
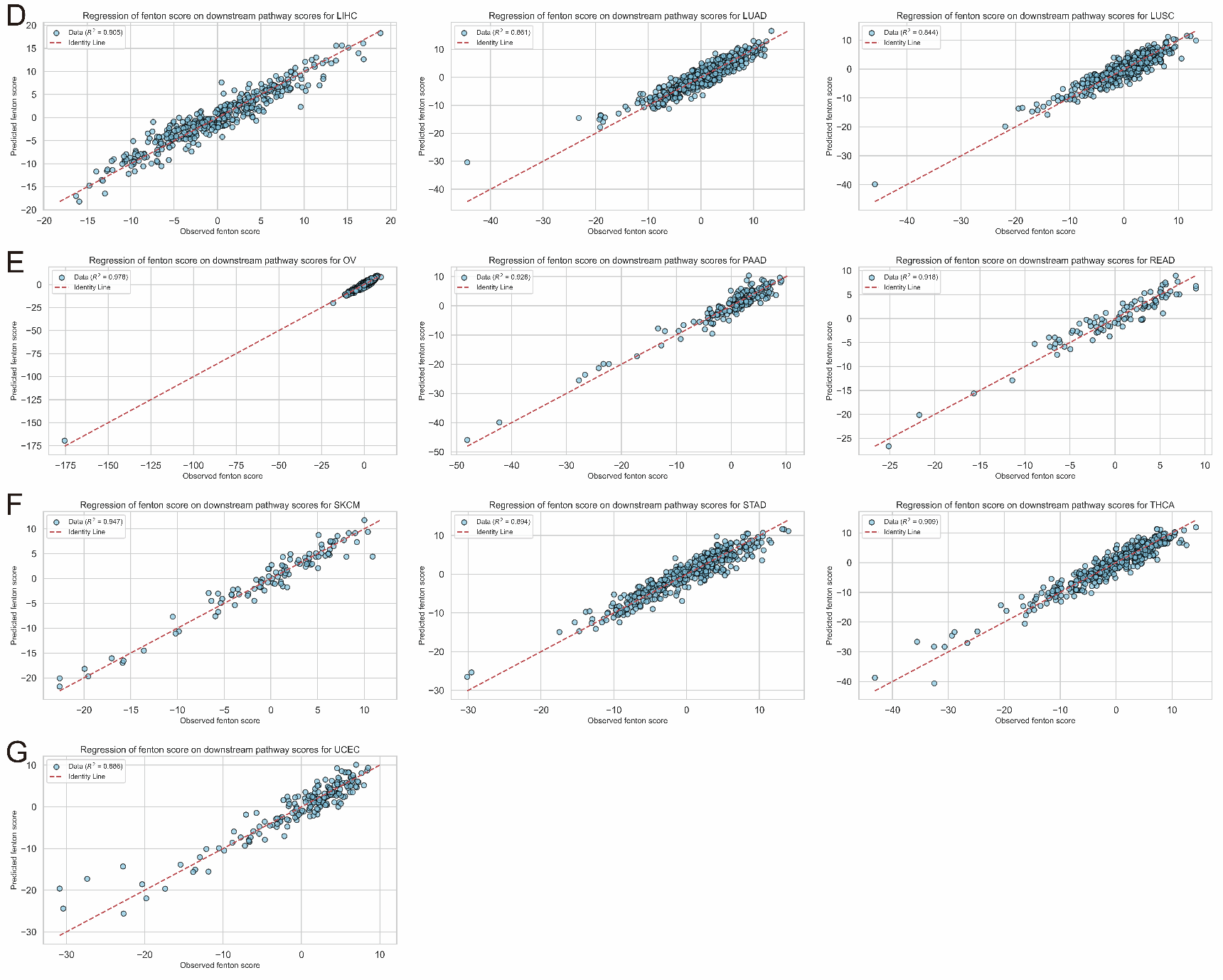
**

**Figure S5:** Regression analyses of the level of Fenton reaction against eight glucose metabolic pathways across 19 cancer types.


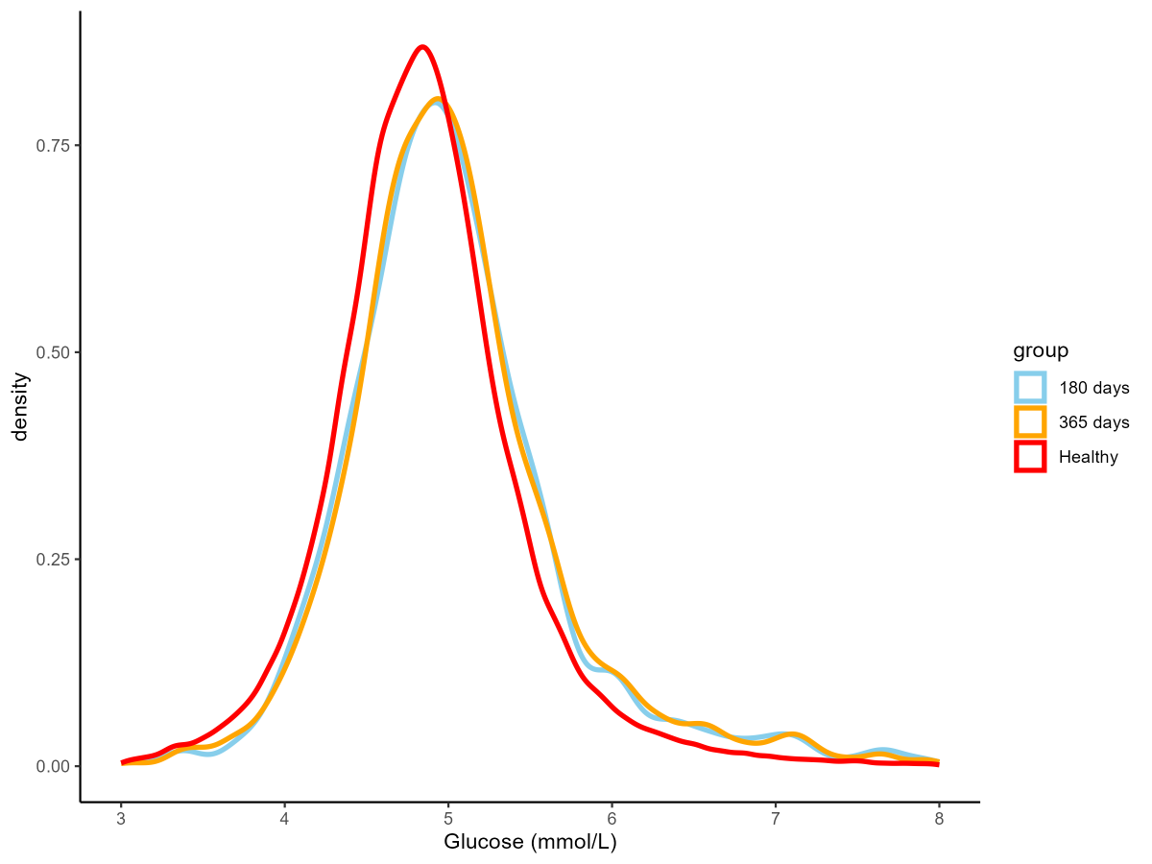


**Figure S6**: Glucose level distributions, where the red, blue and yellow are for the density distractions of glucose levels for heathy people, people detected having developed cancer 180 days and 365 days after the blood test.


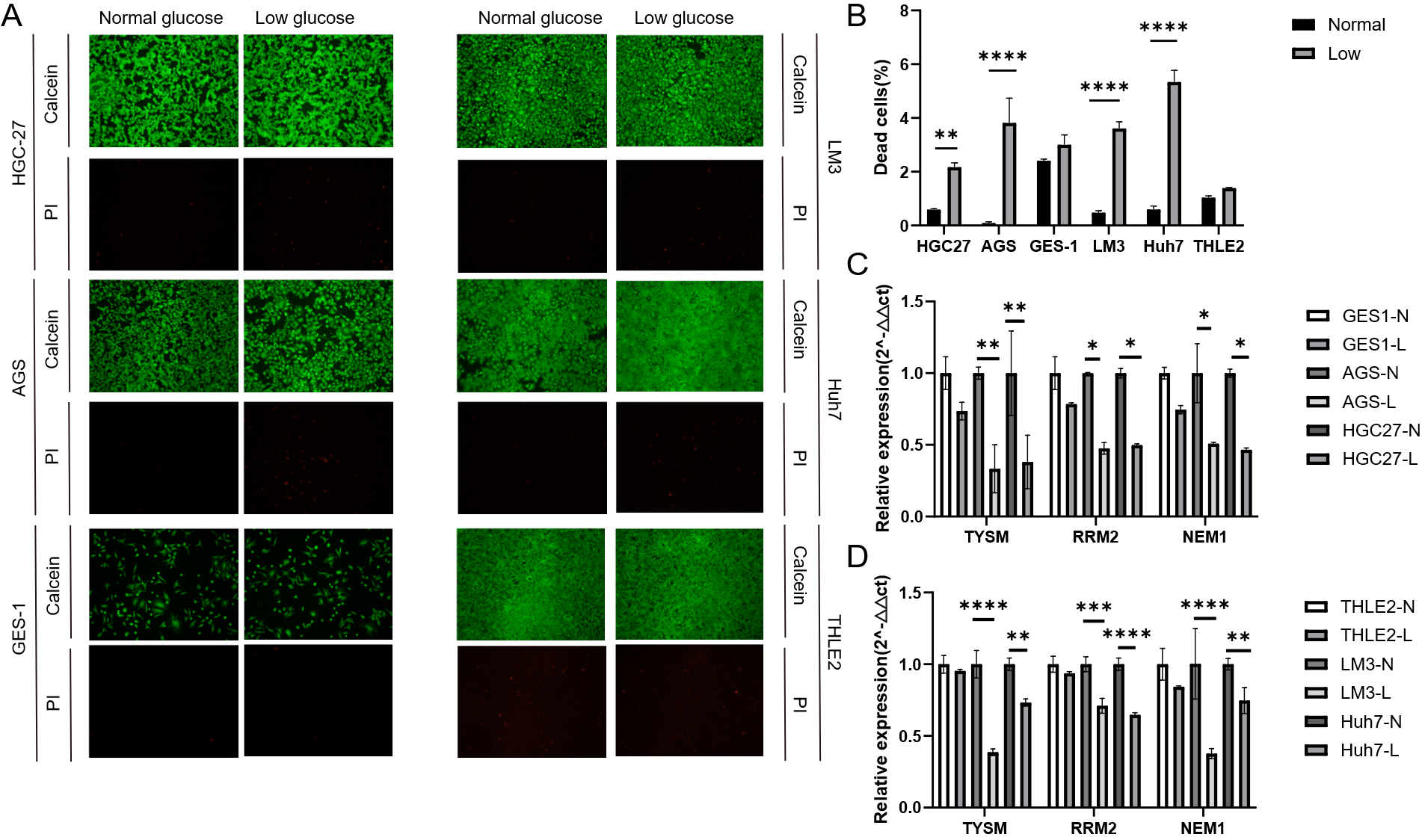


**Figure S7**: Differential viability and gene expression in cancer and normal cell lines under glucose restriction following Fenton reaction-induced alkaline stress. Results demonstrate a significant increase in cell death and downregulation of nucleotide synthesis genes in cancer cell lines under low-glucose conditions, highlighting their glucose dependency compared to normal cell lines.

**Table S1:** An estimate of the level of upregulation of the eight glucose metabolic pathways across different stages of 19 cancer types.

| **Metabolic Process** | **Stage** | **BLCA** | **BRCA** | **CESC** | **COAD** | **ESCA** | **HNSC** | **KICH** | | **KIRC** | **KIRP** | **LIHC** | **LUAD** | **LUSC** | **OV** | **PAAD** | **READ** | **SKCM** | **STAD** | **THCA** | **UCEC** |
| --- | --- | --- | --- | --- | --- | --- | --- | --- | --- | --- | --- | --- | --- | --- | --- | --- | --- | --- | --- | --- | --- |
| **TCA** | **S1** | 0.24 | 0.38 | 0.48 | 0.33 | 0.52 | 0.24 | | 0.48 | 0.05 | 0.33 | 0.76 | 0.52 | 0.71 | 0.57 | 1 | 0.57 | 0.05 | 0.67 | 0.19 | 0.33 |
|  | **S2** | 0.24 | 0.52 | 0.38 | 0.43 | 0.71 | 0.19 | | 0.48 | 0.05 | 0.24 | 0.67 | 0.57 | 0.71 | 0.38 | 1 | 0.29 | 0.62 | 0.57 | 0.29 | 0.29 |
|  | **S3-4** | 0.24 | 0.52 | 0.43 | 0.29 | 0.57 | 0.33 | | 0.38 | 0.14 | 0.24 | 0.71 | 0.71 | 0.67 | 0.67 | 0.81 | 0.38 | 0.52 | 0.62 | 0.14 | 0.29 |
| **Ganglioside synthesis** | **S1** | 0.25 | 0.4 | 0.3 | 0.4 | 0.3 | 0.55 | | 0.25 | 0.5 | 0.45 | 0.55 | 0.5 | 0.55 | 0.25 | 0.95 | 0.4 | 0 | 0.55 | 0.3 | 0.5 |
|  | **S2** | 0.2 | 0.5 | 0.2 | 0.4 | 0.5 | 0.5 | | 0.2 | 0.3 | 0.2 | 0.65 | 0.55 | 0.45 | 0.2 | 0.95 | 0.4 | 0.6 | 0.5 | 0.25 | 0.2 |
|  | **S3-4** | 0.3 | 0.4 | 0.25 | 0.4 | 0.55 | 0.5 | | 0.25 | 0.55 | 0.4 | 0.6 | 0.5 | 0.45 | 0.4 | 0.75 | 0.35 | 0.65 | 0.55 | 0.25 | 0.3 |
| **Glycosylation** | **S1** | 0.46 | 0.62 | 0.41 | 0.6 | 0.51 | 0.6 | | 0.16 | 0.5 | 0.5 | 0.72 | 0.63 | 0.57 | 0.37 | 0.85 | 0.59 | 0.01 | 0.72 | 0.46 | 0.47 |
|  | **S2** | 0.4 | 0.66 | 0.37 | 0.59 | 0.6 | 0.57 | | 0.21 | 0.29 | 0.38 | 0.66 | 0.63 | 0.56 | 0.32 | 0.9 | 0.54 | 0.56 | 0.74 | 0.28 | 0.38 |
|  | **S3-4** | 0.49 | 0.63 | 0.34 | 0.65 | 0.71 | 0.6 | | 0.13 | 0.37 | 0.49 | 0.65 | 0.63 | 0.51 | 0.53 | 0.47 | 0.56 | 0.47 | 0.76 | 0.38 | 0.4 |
| **Lactate production** | **S1** | 0.33 | 0.67 | 0.67 | 1 | 0.33 | 1 | | 0 | 0.33 | 0.33 | 0.67 | 1 | 1 | 0.67 | 0.33 | 0.67 | 0.67 | 0.67 | 0 | 1 |
|  | **S2** | 1 | 0.67 | 0.67 | 0.67 | 1 | 1 | | 0 | 0.33 | 0.33 | 0.67 | 1 | 1 | 0.67 | 0.67 | 0.67 | 1 | 0.67 | 0.33 | 0.67 |
|  | **S3-4** | 0.33 | 0.67 | 0.67 | 0.67 | 1 | 1 | | 0 | 0.33 | 0.33 | 0.67 | 1 | 1 | 0.67 | 0.33 | 0.67 | 1 | 0.67 | 0.33 | 1 |
| **Nucleotide synthesis** | **S1** | 0.5 | 0.71 | 0.6 | 0.75 | 0.5 | 0.73 | | 0.02 | 0.62 | 0.73 | 0.71 | 0.71 | 0.67 | 0.46 | 0.81 | 0.71 | 0 | 0.81 | 0.42 | 0.58 |
|  | **S2** | 0.54 | 0.77 | 0.58 | 0.75 | 0.65 | 0.71 | | 0.06 | 0.31 | 0.56 | 0.73 | 0.69 | 0.62 | 0.46 | 0.88 | 0.79 | 0.69 | 0.81 | 0.38 | 0.48 |
|  | **S3-4** | 0.56 | 0.73 | 0.6 | 0.75 | 0.56 | 0.73 | | 0.02 | 0.54 | 0.75 | 0.77 | 0.73 | 0.65 | 0.58 | 0.75 | 0.75 | 0.65 | 0.81 | 0.42 | 0.48 |
| **One carbon metabolism** | **S1** | 0.45 | 0.45 | 0.5 | 0.55 | 0.36 | 0.73 | | 0.09 | 0.32 | 0.27 | 0.36 | 0.55 | 0.5 | 0.32 | 0.55 | 0.59 | 0.05 | 0.55 | 0.41 | 0.5 |
|  | **S2** | 0.45 | 0.45 | 0.45 | 0.55 | 0.55 | 0.64 | | 0.05 | 0.05 | 0.27 | 0.36 | 0.55 | 0.45 | 0.27 | 0.64 | 0.5 | 0.55 | 0.55 | 0.36 | 0.45 |
|  | **S3-4** | 0.5 | 0.45 | 0.36 | 0.59 | 0.32 | 0.73 | | 0 | 0.14 | 0.41 | 0.27 | 0.55 | 0.45 | 0.41 | 0.32 | 0.59 | 0.41 | 0.55 | 0.36 | 0.5 |
| **Serine synthesis** | **S1** | 0.75 | 0.5 | 1 | 1 | 0 | 0.75 | | 0 | 0.5 | 0.5 | 0.5 | 0.75 | 1 | 1 | 0.5 | 1 | 0 | 0.75 | 0.5 | 0.75 |
|  | **S2** | 0.75 | 1 | 1 | 1 | 0.75 | 0.75 | | 0 | 0.25 | 0.5 | 0.5 | 0.75 | 1 | 0.5 | 0.5 | 0.75 | 0.25 | 0.75 | 0.5 | 0.75 |
|  | **S3-4** | 0.75 | 0.5 | 1 | 1 | 0.25 | 0.75 | | 0 | 0.25 | 0.5 | 0.5 | 0.75 | 1 | 1 | 0.5 | 1 | 0.25 | 0.75 | 0.5 | 0.75 |
| **Sialic acid synthesis** | **S1** | 0.5 | 0.62 | 0.44 | 0.5 | 0.44 | 0.69 | | 0.19 | 0.44 | 0.62 | 0.5 | 0.5 | 0.44 | 0.44 | 0.88 | 0.56 | 0 | 0.62 | 0.25 | 0.56 |
|  | **S2** | 0.5 | 0.62 | 0.38 | 0.56 | 0.5 | 0.56 | | 0.25 | 0.31 | 0.31 | 0.44 | 0.56 | 0.44 | 0.31 | 0.81 | 0.5 | 0.62 | 0.62 | 0.19 | 0.31 |
|  | **S3-4** | 0.56 | 0.62 | 0.44 | 0.5 | 0.5 | 0.62 | | 0.19 | 0.5 | 0.56 | 0.5 | 0.62 | 0.5 | 0.44 | 0.56 | 0.56 | 0.56 | 0.62 | 0.12 | 0.38 |
| **Sphingolipid synthesis** | **S1** | 0.27 | 0.5 | 0.3 | 0.45 | 0.38 | 0.45 | | 0.2 | 0.32 | 0.38 | 0.52 | 0.41 | 0.41 | 0.39 | 0.8 | 0.39 | 0 | 0.61 | 0.38 | 0.43 |
|  | **S2** | 0.25 | 0.52 | 0.25 | 0.48 | 0.52 | 0.43 | | 0.21 | 0.16 | 0.2 | 0.55 | 0.38 | 0.43 | 0.27 | 0.88 | 0.38 | 0.45 | 0.61 | 0.29 | 0.23 |
|  | **S3-4** | 0.29 | 0.5 | 0.21 | 0.46 | 0.5 | 0.43 | | 0.16 | 0.3 | 0.27 | 0.55 | 0.34 | 0.39 | 0.5 | 0.54 | 0.38 | 0.34 | 0.61 | 0.32 | 0.32 |
| **Triglyceride synthesis** | **S1** | 0.36 | 0.29 | 0.29 | 0.36 | 0.29 | 0.29 | | 0.14 | 0.29 | 0.21 | 0.57 | 0.5 | 0.14 | 0.29 | 0.57 | 0.29 | 0.07 | 0.43 | 0.29 | 0.29 |
|  | **S2** | 0.36 | 0.29 | 0.29 | 0.21 | 0.29 | 0.14 | | 0.14 | 0.21 | 0.14 | 0.5 | 0.36 | 0.21 | 0.43 | 0.71 | 0.29 | 0.29 | 0.57 | 0.29 | 0.29 |
|  | **S3-4** | 0.36 | 0.29 | 0.21 | 0.36 | 0.36 | 0.36 | | 0 | 0.36 | 0.14 | 0.5 | 0.43 | 0.14 | 0.57 | 0.5 | 0.29 | 0.21 | 0.5 | 0.29 | 0.29 |
| **Unsaturated fatty acid synthesis** | **S1** | 0.33 | 0.33 | 0.33 | 0.67 | 0 | 0.33 | | 0 | 0 | 0.33 | 0.67 | 0.33 | 0.33 | 0.33 | 0.67 | 0.67 | 0 | 0.67 | 0 | 0.67 |
|  | **S2** | 0.33 | 0.33 | 0.67 | 0.67 | 0.33 | 0.33 | | 0.33 | 0 | 0.33 | 0.33 | 0.33 | 0.33 | 0.33 | 0.67 | 0 | 0 | 0.33 | 0 | 0.67 |
|  | **S3-4** | 0.33 | 0.33 | 0.67 | 0.67 | 0 | 0.33 | | 0 | 0 | 0.67 | 0.67 | 0.33 | 0.33 | 0.33 | 0.33 | 0.67 | 0 | 0.67 | 0 | 0.67 |

where Sk is for stage k, 1 $\leq k \leq4$; each value in the table represents the number of upregulated genes in cancer vs. control tissues divided by the total number of genes in the corresponding pathway.
